# Pathophysiology and preclinical relevance of experimental graft-versus-host disease in humanized mice

**DOI:** 10.1186/s40364-024-00684-9

**Published:** 2024-11-14

**Authors:** Grégory Ehx, Caroline Ritacco, Frédéric Baron

**Affiliations:** 1 Laboratory of Hematology, GIGA Institute, University of Liege, Liege, Belgium; 2 Walloon Excellence in Life Sciences and Biotechnology (WELBIO) Department, WEL Research Institute, Wavre, Belgium; 3 Department of Medicine, Division of Hematology, CHU of Liege, University of Liege, Liege, Belgium

**Keywords:** GVHD, Xenogeneic, NSG mice, Hematopoietic cell transplantation

## Abstract

Graft-versus-host disease (GVHD) is a life-threatening complication of allogeneic hematopoietic cell transplantations (allo-HCT) used for the treatment of hematological malignancies and other blood-related disorders. Until recently, the discovery of actionable molecular targets to treat GVHD and their preclinical testing was almost exclusively based on modeling allo-HCT in mice by transplanting bone marrow and splenocytes from donor mice into MHC-mismatched recipient animals. However, due to fundamental differences between human and mouse immunology, the translation of these molecular targets into the clinic can be limited. Therefore, humanized mouse models of GVHD were developed to circumvent this limitation. In these models, following the transplantation of human peripheral blood mononuclear cells (PBMCs) into immunodeficient mice, T cells recognize and attack mouse organs, inducing GVHD. Thereby, humanized mice provide a platform for the evaluation of the effects of candidate therapies on GVHD mediated by human immune cells in vivo. Understanding the pathophysiology of this xenogeneic GVHD is therefore crucial for the design and interpretation of experiments performed with this model. In this article, we comprehensively review the cellular and molecular mechanisms governing GVHD in the most commonly used model of xenogeneic GVHD: PBMC-engrafted NOD/LtSz-Prkdc^scid^IL2rγ^tm1Wjl^ (NSG) mice. By re-analyzing public sequencing data, we also show that the clonal expansion and the transcriptional program of T cells in humanized mice closely reflect those in humans. Finally, we highlight the strengths and limitations of this model, as well as arguments in favor of its biological relevance for studying T-cell reactions against healthy tissues or cancer cells.

## Introduction

Graft-versus-host disease (GVHD) is a life-threatening complication of allogeneic hematopoietic cell transplantations (allo-HCT). Allo-HCT is a commonly used treatment option where the patient’s hematopoietic and immune systems are replaced by healthy hematopoietic cells derived from a suitable donor. It is used to treat either congenital or acquired disorders, such as hematological malignancies, with acute myeloid leukemia being the most common indication for allo-HCT.

Typically, the allo-HCT procedure can be summarized in three steps: the conditioning regimen, the transplantation itself, and the immune reconstitution. The conditioning regimen consists of chemotherapy combined or not with radiotherapy. When allo-HCT is given as treatment of hematological malignancies, the aims of the conditioning regimen are: (i) the reduction or eradication of the malignant cells, (ii) the clearance of patient bone marrow (BM) niches to allow the engraftment of donor hematopoietic cells and (iii) the suppression of the host immune system to prevent graft rejection. Following the conditioning regimen, patients receive the infusion of the donor hematopoietic cells. Whereas the grafts contain hematopoietic progenitor and stem cells, they also contain a significant number of mature immune cells, including T cells. These T cells play a pivotal role in the graft-versus-leukemia (GVL) effects, through which donor T cells eradicate the remaining malignant cells having survived the conditioning regimen, thereby preventing disease relapse. Unfortunately, transplanted donor immune cells are also susceptible to recognizing and targeting healthy organs from the recipient causing GVHD. Despite the availability of multiple treatments aiming at preventing it, 30 to 70% of recipients develop some form of GVHD, and up to 30% of cases eventually result in the death of the patient [1].

The understanding of GVHD pathophysiology has made tremendous progress thanks to the usage of murine models of allo-HCT [2]. However, due to fundamental differences in murine and human immunology, these models have some limitations. Recently, the usage of humanized mouse models of GVHD has circumvented several of these limitations. In the present review, we will first provide a general overview of GVHD immunobiology and briefly introduce the conventional mouse-to-mouse models of allo-HCT. We will then compare these models to the most recent humanized mouse model of GVHD and detail the mechanisms of GVHD pathophysiology in these animals. Finally, we will discuss their usage for the validation of novel therapeutic options of GVHD as well as discuss the limitations inherent to this model.

## Immunobiology of GVHD

GVHD is typically classified into two distinct syndromes depending in part on the time of its occurrence after transplant. Acute GVHD (aGVHD) is defined as an inflammatory process occurring early (i.e. in the first months) after transplantation and involving the skin, liver, and/or gastrointestinal tract [2]. However, late acute GVHD can also occur, especially in patients given grafts after nonmyeloablative conditioning [3]. In contrast, chronic GVHD (cGVHD) most often occurs later and shares clinical features with autoimmune disorders such as scleroderma, systemic lupus erythematosus, sicca syndrome, sclerosing cholangitis, and/or lung transplant rejection (bronchiolitis obliterans). In the present review, we will focus on acute GVHD as humanized mouse models better mimic this type of GVHD.

One of the main predictors of GVHD development is the extent of HLA disparity between the donor and the host. In humans, this degree of HLA mismatching is directly related to the frequency and severity of GVHD [4]. The best suitable donor to prevent GVHD development is therefore an identical twin. However, few patients have an HLA-identical twin and the usage of such donors dramatically increases the risk of relapse [5], as the efficacy of the GVL effect depends on the level of genetic disparities (minor or major histocompatibility antigen mismatches) between the immune cells of the donor and the leukemic cells of the recipient. In addition, a lower incidence of relapse is observed in recipients experiencing GVHD, evidencing a desirable effect of mild GVHD on transplantation outcome [3, 5]. Transplantation settings characterized by the presence of genetic disparities between donor and recipient are therefore favored for the treatment of patients with hematological malignancies. Therefore, GVHD (which occurs in up to 70% of patients [1]) needs to be managed with prophylaxis and treatments. Currently, GVHD prophylaxis includes immunosuppressive agents such as calcineurin inhibitors (Cyclosporin-A and Tacrolimus), anti-metabolites (Mycophenolate Mofetil (MMF) and Methotrexate, MTX), post-transplant cyclophosphamide (PTCy), T-cell depleting antibodies (Antithymocyte globulins (ATG), and Abatacept (a recombinant soluble CTLA-4-Ig) [6]. The first-line treatment option is the use of corticosteroids such as prednisolone and methylprednisolone while the second line is Ruxolitinib [7]. Third-line and experimental GVHD treatment options were extensively reviewed elsewhere [8].

T cells are the key players in GVHD. Indeed, ex vivo T-cell depletion from the graft reduces dramatically the incidence of GVHD even without post-grafting immunosuppression [9] At the cellular level, donor T cells are mainly activated by recipient antigen-presenting cells (APCs) through the recognition of MHC-associated peptides presented by APCs by the T-cell receptor (TCR) of T cells. Importantly, in experimental mouse models of GVHD as well as in patients after myeloablative conditioning regimens, host APCs are themselves activated by the release of damage-associated molecular patterns (DAMPs), pathogen-associated molecular patterns (PAMPs), and proinflammatory cytokines (TNF-α, IL-1, IL-6, CCL2/3/4/5, CXCL10/11) subsequent to tissue damages induced by the conditioning regimen (cytokine storm) [10, 11]. Furthermore, the intestinal microbiota diversity, which may influence the nature and amount of PAMPs released following intestinal injury by the conditioning regimen, is increasingly considered pivotal in GVHD pathophysiology [12, 13]. Following their activation, host APCs increase their expression of MHCs, chemokines, adhesion molecules, and co-stimulatory molecules (that will provide co-stimulation to T cells through CD80/CD86 signaling) [14]. However, severe GVHD can also occur in the absence of a cytokine storm, as demonstrated by the high incidence of GVHD observed in patients given donor lymphocyte infusions (DLI) as treatment for post-transplant relapses [15]. Subsequently, the mechanism of T-cell activation is different between matched and mismatched allo-HCT.

In MHC-matched HCT, immunogenic alloantigens presented by MHC molecules are mostly endogenous minor histocompatibility antigens (peptides generated by polymorphic genes differing between donor and host) [16, 17]. Exogenous antigens (acquired by APCs via phagocytosis of dead or necrotic cells, endocytosis, or macropinocytosis) can also be presented within MHC-I by cross-presentation [18]. In contrast, alloantigens presented by MHC-II molecules are exogenous minor antigens. Consequently, alloantigens may be presented by MHC-II molecules from both recipient or donor APCs.

In MHC-mismatched HCT, donor T cells can cross-react to host mismatched MHC alleles loaded with an antigenic peptide through a process known as molecular mimicry, violating the paradigm of self-MHC restriction [19–23]. Specifically, host peptide-MHC complexes (loaded with peptides that are either allogeneic or not) are capable of engaging a specific donor TCR clone by adopting a three-dimensional conformation similar to the cognate peptide-MHC complex of the clone (a phenomenon predominant within the virus-specific T-cell population characterized by high affinity for their cognate peptide-MHC complex [24, 25]). Further, the TCR itself may also undergo conformational “fine-tuning” to accommodate minor conformational alterations in peptide-MHC complexes. The molecular aspects of these mechanisms have been extensively reviewed by Gras et al. [26] and Smith et al. [27]. Considering the high plasticity of these mechanisms, illustrated by the high frequency of T cells reacting to MHC-mismatched allogeneic APCs in vitro (1–10%) [28, 29], they may be responsible for the high incidence of GVHD when transplanting across multiple MHC mismatches (with growing numbers of mismatched loci increasing the probability of allogeneic reactions). Nevertheless, alloreactive T cells can also retain their capacity to recognize specifically their cognate peptide antigen, presented by allogeneic MHC alleles [30]; allogeneic reactions should therefore not be only considered unspecific.

After activation by host APCs, the majority of transplanted T cells present a memory or activated phenotype, resulting mostly from the activation of naive (CD45RA^+^CD62L^+^) T cells and the expansion of their mature (CD45RA^─^) counterparts. Reflecting the presumed antigenic specificity of the allogenic reactions, the diversity of TCR specificities repertoire (indirectly reflecting the diversity of cognate MHC antigens) of transplanted T cells dramatically shrinks after transplantation (higher oligoclonality) [31]. This results notably from the fact that the naive T cells present a greater TCR repertoire diversity than antigen-experienced memory T cells [32]. Naive T cells remain profoundly depleted for many months after transplantation and the expansion of T cells post-HCT is often characterized by the dominance of oligoclonal T cell populations [33]. Thereby, the restoration of a diverse T-cell population depends mainly on the generation of T-cell progenitors by donor hematopoietic stem cells [34]. Interestingly, a greater oligoclonality has been associated with GVHD development in patients following HCT, suggesting that the GVH reaction may depend on a limited set of MHC antigens [35–40].

To achieve proper activation-induced proliferation, T cells require the combination of TCR stimulation and co-stimulation (CD80/CD86) signals but also require the signaling of cytokines. Among them, the interleukin-2 (IL-2) and its receptor components play a pivotal role in the initiation of the exponential proliferation of T cells [41]. Thereby, IL-2 has long been considered a key player in GVHD aggravation as treatments inhibiting its expression (such as calcineurin inhibitors) successfully mitigate GVHD. However, more recent findings evidenced that IL-2 also non-redundantly sustains the proliferation of regulatory T cells (Tregs), immunotolerant cells able to mitigate experimental GVHD [42, 43]. Therefore, IL-2 and its dual role in GVHD is under intense investigation, and therapies modulating IL-2 levels toward a better promotion of Tregs have shown some success in patients receiving allo-HCT [44]. In addition to IL-2, many other cytokines (IL-4, IL-7, IL-9, IL-15, and IL-21), secreted either by immune or stromal cells, further participate in T-cell proliferation. However, IL-7 and IL-15 in particular are considered key players in GVHD as they provide critical signals to drive T cell proliferation in the lymphopenic conditions following the conditioning regimen and are predictors of GVHD development [45–47]. In particular, a recent study evidenced that the IL-7 receptor signaling drives the pathologic damages mediated by CD4^+^ T cells in the gastrointestinal tract [48]. In contrast, administration of IL-7 did not induce GVHD in patients given T-cell depleted grafts [49].

Besides their shift toward a memory/activated phenotype following activation by APCs, T cells also undergo differentiation into specialized effector subsets. While naive T cells can differentiate into a myriad of different effectors (for a detailed review see [50]), it is commonly admitted that naive CD4^+^ T cells can differentiate into three major pro-inflammatory subsets: Th1, Th2, and Th17. Among them, the Th1 subset is usually considered to be the main mediator of GVHD as they secrete abundant amounts of IFN-γ and TNF-α, two cytokines mediating direct tissue damages as well as increasing the recruitment of other T cells and increasing the inflammatory process [11, 51–53]. Th17 is the second most studied inflammatory T-cell subset in GVHD. While their function in aGVHD remains a matter of debate [54–56], their characteristic secretion of IL-17 plays a major role in the promotion of tissue inflammation [57] and they are instrumental in cGVHD pathogenesis [58, 59]. Similarly, the role of Th2 in GVHD is also controversial, with studies suggesting that they either aggravate [60] or ameliorate [61] GVHD. Reflecting helper T cells, cytotoxic T cells also differentiate into multiple subsets, including Tc1, Tc2, and Tc17, the latter having been particularly linked to GVHD [62, 63].

Finally, differentiated T cells progressively leave the secondary lymphoid organs (where they mainly home after infusion [64]) and migrate to peripheral organs, the targets of GVHD [65]. This trafficking is notably driven by the loss of naive phenotype (characterized by the expression of homing receptors to enter secondary lymphoid tissues, such as CD62L) and the upregulation of several chemokine receptors. CXCR3 [66, 67], CCR2 [68, 69], CCR5 [70, 71], CCR6 [72, 73], CLA [74], and α4β7 [75] integrin expression by T cells are notably involved in this trafficking. In peripheral organs, T cells induce tissue damage through multiple mechanisms including the release of effector cytokines (mentioned above), the production of lytic enzymes (granzyme and perforin) [76], and through the Fas/FasL pathway [76]. These damages then participate in the aggravation of inflammation, leading to the recruitment of additional immune cells, thereby inducing a feedback loop that may be responsible for organ failure if not treated.

## Humanized NSG mice as models of GVHD

Currently, most of our understanding of GVHD pathophysiology is based on murine models of allo-HCT. These models usually involve transplanting BM (as a source of hematopoietic stem cells), supplemented with varying numbers and types of donor lymphocytes, into irradiated allogeneic recipients that differ from the donors in their MHC (with various extents of MHC mismatch between the donor and recipient). Thereby, such models mimic well the clinical setting of myeloablative allo-HCT (high-dose conditioning followed by allogeneic cell transplantation) and they successfully enabled the identification of several anti-GVHD molecules such as JAK inhibitors [77]. However, these models also suffer from inherent limitations (listed in Table 1) such as fundamental differences between human and mouse immunology [78], the fixed genetic disparity between donor and recipient, the use of young donors/recipients, and the homogenous microbial environment since mice are bred under pathogens-free conditions [79, 80]. Therefore, a pre-clinical model allowing the study of GVHD mediated by human T cells and enabling the use of donors with various genetics, ages, and exposure to pathogens is a strong complement to strictly murine-based models (Table 1). Such a model requires highly immunodeficient mice capable of engrafting functional human cells or tissues without rejecting them.

**Table 1:** A point-by-point comparison of the pros and cons of humanized mice (immunodeficient mice engrafted with human PBMCs) vs. conventional mouse-to-mouse transplantation models for the study of GVHD

Humanized mice	Conventional mouse models
**CON**: poorly representative of the human clinical setting (transplantation of PBMCs and xenoreactions).	**PRO**: closer to the human clinical setting (transplantation of hematopoietic stem cells + mature immune cells and alloreactions).
**CON**: limited genetic engineering of the graft, and of the recipient.	**PRO**: virtually unlimited genetic engineering of the graft and the recipient through the usage of the broadly available mutant mouse strains.
**PRO**: GVHD mediated by human cells	**CON**: GVHD mediated by mouse cells
**PRO**: possibility to use PBMCs from any donor, allowing the reproduction of the genetic diversity of the donors in the clinic.	**CON**: fixed genetic diversity between donor and recipient, poorly representative of the human clinical setting.
**PRO**: irradiation is not necessary for engraftment, enabling the study of GVHD independently of the pro-inflammatory conditions induced by this regimen. Chemotherapy-based conditioning can also be performed (busulfan).	**CON**: engraftment requires high doses of irradiation, poorly representative of the human clinical setting in which many patients nowadays receive chemotherapy-based reduced-intensity conditioning.
**PRO**: possible to use primary human leukemic cells to study the GVL effect.	**CON**: usage of a limited number of malignant cell lines, with low clonal heterogeneity.
**PRO**: usage of PBMCs from donors of any age, previously exposed to real-life immunological conditions (past infections, auto-immune diseases, …).	**CON**: graft obtained from young animals, housed in pathogen-free conditions.
**CON**: mice are more expensive and have to be kept protected from pathogens anytime.	**PRO**: mice are cheap and require less expensive housing conditions.
**PRO**: a single mouse strain is needed (e.g. NSG).	**CON**: two strains (e.g. BALB/c and C57BL/6) are necessary, raising the costs and space of housing.
**PRO**: limited number of experimental steps to transplant animals, reducing the time and workload needed for transplantation.	**CON**: greater number of experimental steps (including the sacrifice/dissection/sorting of cells from donor mice).
**CON**: absence of some key cytokines involved in GVHD pathogenesis (such as IL-7 and IL-15).	**PRO**: presence of all cytokines involved in GVHD pathogenesis.
**CON**: possibly limited contribution of non-hematopoietic APCs to GVHD pathogenesis.	**PRO**: non-hematopoietic APCs contribute to GVHD pathogenesis.
**PRO**: expected response to GVHD-mitigating drugs validated in the clinic.	**PRO**: expected response to GVHD-mitigating drugs validated in the clinic.
**CON**: lymph nodes are underdeveloped and poorly or not playing their role in T-cell priming.	**PRO**: all organs involved in T-cell priming are present.

The first step in the development of immunodeficient mice came with the discovery of the Prkdc^scid^ mutation (protein kinase, DNA activated, catalytic polypeptide; severe combined immunodeficiency). This mutation occurred spontaneously in a colony of CB-17 mice housed in the Institute for Cancer Research in Philadelphia in 1983 [81] and is responsible for the creation of a premature stop codon in the amino acids sequence of Prkdc. Subsequently, the translation of the Prkdc protein, which has a critical role in V(D)J segment recombination, is substantially reduced in scid mice, impairing T- and B-cell development. The description of the scid mutation was soon followed by the observation that human mature immune cells [82] and hematopoietic stem cells [83] could engraft in these mice. However, human cell engraftment was limited by the high levels of host NK cells, the activity of myeloid lineage cells, and the spontaneous generation of T and B cells during aging (a phenomenon known as leakiness).

A breakthrough came with the backcrossing of the scid mutation onto the non-obese diabetic (NOD) background [84]. The NOD strain is a polygenic model for spontaneous autoimmune type 1 diabetes. NOD mice are characterized by multiple aberrant immunophenotypes including defective antigen-presenting cells, defects in the regulation of the T-cell repertoire, defective NK cell function, defective cytokine production by macrophages, and a lack of hemolytic complement, C5. Importantly, NOD mice present a polymorphism in the Sirpa gene (encoding the signal regulatory protein-α, SIRP-α) which renders it very similar to the human gene [85]. Therefore, appropriate interaction between SIRP-α on host macrophages with the human CD47 expressed by engrafted hematopoietic cells can act as an inhibitory signal preventing the phagocytosis of human cells by murine macrophages. The combination of these properties and the effects of the scid mutation in NOD-scid mice allowed reaching frequencies of peripheral circulating human cells between 1 and 10% 85, 86. However, this model remained limited by the short life span (due to the development of lethal thymic lymphomas) of mice and the residual activity of host NK cells.

The last breakthrough in the field of immunodeficient mice has been the development of mice homozygous for targeted mutations of the interleukin-2 receptor γ-chain locus (Il2rg), also known as the common gamma chain (γc, or CD132) [87–90]. This mutation results in severe impairment of T- and B cells, complete prevention of NK cell development, absence of leakiness, and absence of spontaneous lymphoma development. The NOD-scid IL-2Rγ^−/−^ mice have been developed by two distinct teams, creating the NSG mice [91] (in which the IL-2Rγ is completely absent) and the NOG mice [87] (in which only the intracytoplasmic tail of the receptor is truncated, preventing its signal transduction). In both strains, the immunological features of IL-2Rγ^−/−^ are combined with the features of NOD-scid mice, resulting in mice in which T-, B- and NK cells are absent in addition to a deficit in complement, macrophages, and dendritic cell function. Upon transplantation of human PBMCs, these mice develop a xenogeneic GVHD, with engraftment success rates reaching virtually 100%^93^. Because they are nearly identical [93] and show similar T-cell engraftment and disease development [94], we will consider NSG and NOG mice as identical (commonly referred to as NSG for NOD-scid IL-2Rγ^−/−^) for the rest of this review, and NSG mice transplanted with human PBMCs will be considered as “humanized”.

In terms of GVHD research, humanized mice offer multiple advantages over conventional mouse-to-mouse transplantation models, the main being the usage of human-derived grafts, representative of those used in the clinical setting (Table 1). However, conventional models also have their advantages, as the allogeneic (in contrast to xenogeneic in NSG mice) reactions taking place in these models better mimic in theory those happening in patients receiving allo-HCT. Therefore, multiple efforts were made to develop a humanized mouse model of allogeneic reactions, notably through the usage of NSG mice transgenic for HLA-A02 [95] or HLA-DR4 [96]. However, the interest in such “allogeneic” models remained limited because of the presence of murine MHC (preservation of xenogeneic reactions). While such xenogeneic reactions could still be good proxies for the study of allogeneic reactions taking place in patients (further discussed herein), the recent design of NSG mice null for murine MHC and transgenic for human MHC [97] represents an exciting progression in the modelization of human allo-HCT in mice. Nevertheless, the NSG mice still represent the most broadly used experimental model to study GVHD mediated by human T cells. Therefore, in the following sections, we will detail the molecular and cellular mechanisms governing the xenogeneic GVHD taking place in NSG mice and will discuss the relevance of these mechanisms regarding human GVHD.

## Pathophysiology of GVHD in humanized mice

### Protocol and clinical signs

Humanized NSG mice are relatively easy to use. As reported in the first article describing the model, GVHD can be induced in NSG mice by a single intravenous injection of low numbers (0.5-5 × 10^6^, most often ~ 2.5 × 10^6^) of human PBMCs following sub-lethal total body irradiation (TBI, 2 Gy) [92, 98–101]. Typically, these PBMCs are obtained directly following gradient isolation from buffy coats or peripheral blood of healthy volunteers. Thereby, the model requires fewer steps of graft manipulation, in contrast to conventional mouse-to-mouse models which typically require the preparation of two cell fractions (T-depleted BM + splenocytes, obtained from healthy mice sacrificed on the day of transplantation). While this low graft manipulation could result in a better inter-lab and intra-lab reproducibility of the model a priori, it is typically characterized by variable GVHD dynamics from experiment to experiment, mostly attributable to (1) the usage of different PBMC donors, mimicking the variable GVHD severity in human patients; (2) the usage of different TBI and PBMC doses; and (3) variabilities in the source and preparation methods of PBMCs (24 h-old buffy coats, fresh blood, cryopreserved PBMCs, cultured PBMCs, …). In addition, GVHD can also be induced without TBI through the transplantation of higher amounts (1–2 × 10^7^) of PBMCs [92, 102, 103], either through i.v. or i.p. injection routes. While the injection route has a relatively low impact on GVHD and engraftment dynamics [98, 104], the usage of TBI (at equal PBMC doses) accelerates the development of GVHD and improves the engraftment rate of human cells [92, 98, 104]. Thereby, the variable usage of TBI across studies further increases the inherent variability of the model and its usage will be considered the principal analyzable source of variation for the rest of the present review.

Clinically, GVHD manifestations in NSG mice include weight loss, hunching, anemia, and mobility loss. Death typically occurs within 20–50 days post-transplantation [92, 94, 95]. Typical symptoms of acute GVHD in humans such as jaundice, diarrhea, and skin rash are rarely observed. Thereby, humanized NSG mice only partially reflect human aGVHD. Nevertheless, many lines of evidence suggest that human T cells attack the liver, gut, and skin (the three organs from which these symptoms derive) in NSG mice: (i) histopathological analyses demonstrated infiltration of human T cells in the liver, colon, and skin [105, 106]; (ii) human T cells found in peripheral blood express high levels of the cutaneous lymphocyte antigen (CLA) as soon as 7 days post-transplantation [102]; (iii) mice surviving the acute phase of the disease develop signs of chronic GVHD, including hair loss and skin fibrosis [100, 103], (iv) GVHD clinical progression correlates with an aggravation of histopathological damages observed in skin and colon [105], and (v) transplanted mice present signs of hepatic dysfunction such as elevated serum concentration of alanine transaminase and aspartate transaminase [94]. In addition to skin, liver, and colon, human T-cell infiltrations were observed in BM, esophagus, stomach, jejunum, duodenum, rectum, heart, spleen, lung, pancreas, kidney, thyroid, adrenal gland, and skeletal muscles [92, 95, 106]. However, the most important T-cell infiltrations are found in the BM, spleen, liver, and lungs. Because the skin is the most commonly affected organ in human aGVHD, efforts have been made to develop a model that better mimics skin symptoms. Indeed, Ito et al. have reported that skin inflammations including alopecia, epidermal hyperplasia, and neutrophilia can be induced by transplanting only the CD4^+^ T-cell fraction of PBMCs to NOG mice [107]. The molecular details of T-cell homing to peripheral organs will be further discussed in the following sections.

### Early events post-transplantation

**Fig. 1:**
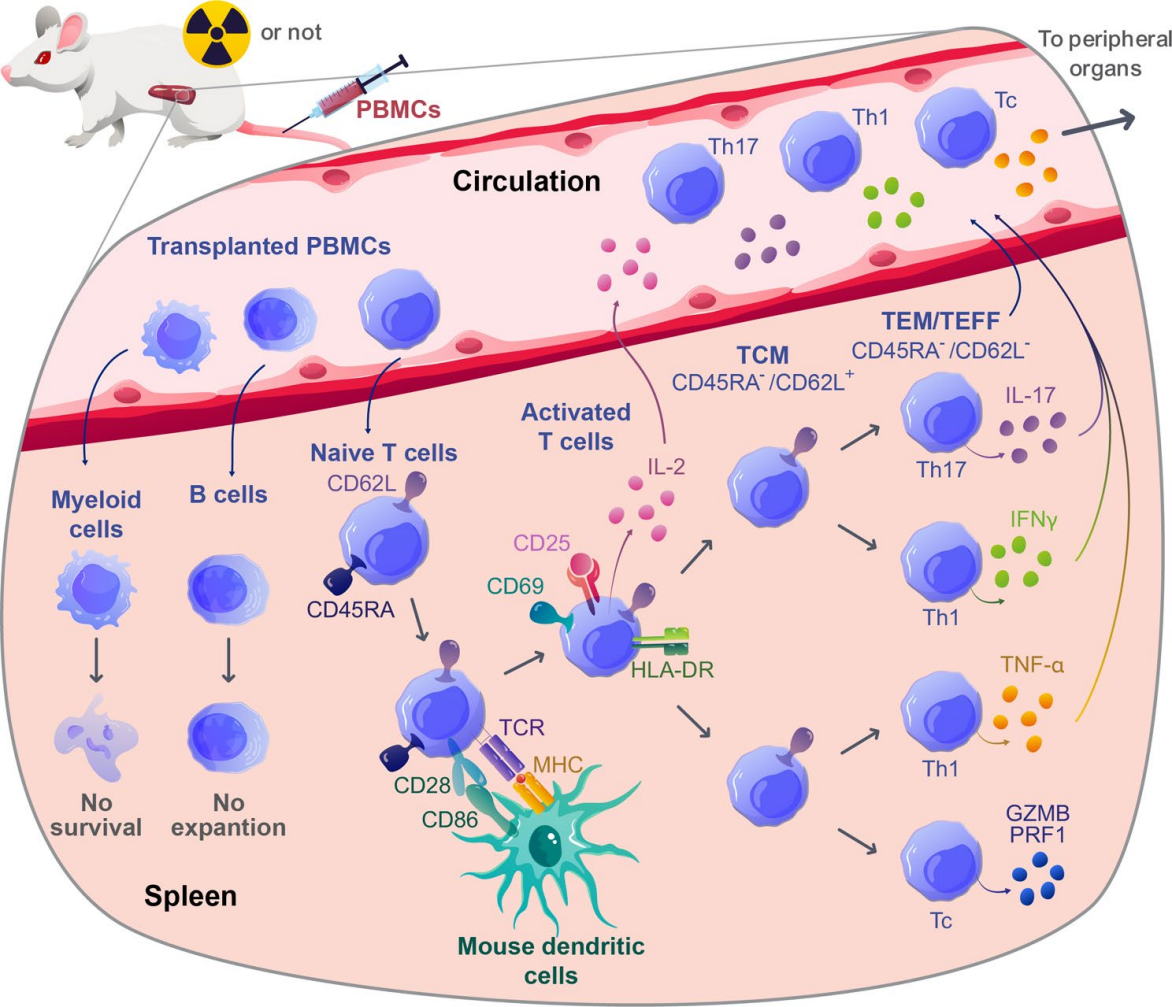
Illustration of the key steps involved in the activation and initial expansion of T cells in NSG mice. After transplantation of PBMCs, only T cells expand in the spleen as some B cells can be detected but do not expand and no myeloid cells can be detected. T cells are then activated by mouse dendritic cells, proliferate, differentiate into TEM/TEFF, and migrate toward peripheral organs. Th1, T helper 1; Th17, T helper 17; Tc, T cytotoxic; TCM, central memory T cells; TEM, Effector-memory T cells; TEFF, effector T cells; GZMB, Granzyme B, PRF1, Perforin-1

Due to their deficiency in γc receptors, the organogenesis of lymph nodes (LNs), Peyer’s patches (PPs), and germinal centers are impaired in NSG mice [108–111]. Therefore, these mice are characterized by poor activation and proliferation of donor B cells upon PBMC injection [94]. Nevertheless, B cells [102] as well as significant levels of antibodies [98] remain observable in the spleen and peripheral blood, respectively, of transplanted animals for several weeks post-transplantation. In addition, due to the low homology between human and mouse cytokines necessary for the myeloid compartment survival (such as GM-CSF, IL-3, FLT3L, CSF, and SCF), the transplanted myeloid cells fail to survive in NSG mice [94, 112–114]. Finally, human NK cells also poorly engraft in NSG mice [94]. This mainly results from their need for IL-15 to sustain their proliferation [115] and the absence of this cytokine in NSG mice. Indeed, the murine IL-15 is inadequate to support human NK cell proliferation [116], and the only transplanted cells able to secrete IL-15 are monocytes / macrophages [117], which do not survive after transplantation. Interestingly, a recent study demonstrated increased NK lytic activity in response to an IL-15 superagonist in humanized NSG mice, suggesting that the lack of IL-15 is indeed responsible for the poor survival of NK cells [118]. Therefore, T cells are the main human cell population to expand in humanized NSG mice, and they are necessary and sufficient for GVHD development (Fig. 1) [92, 94, 104].

Similarly to what has been observed in mouse-to-mouse models of allo-HCT [16, 119], donor APCs are not required for the development of GVHD in NSG mice. Indeed, a comprehensive mechanistic study showed that the removal of donor APCs from the graft did not provide a survival advantage in comparison to the whole PBMCs [104]. Furthermore, by using BM chimeras, the same study showed that host hematopoietic, but not nonhematopoietic, APCs are necessary for the induction and development of GVHD. However, this study did not use TBI before the transplantation of PBMCs to chimeras, and therefore the role of non-hematopoietic APCs (which can act as GVHD-initiating cells when activated by the conditioning regimen [120]) in GVHD initiation could have been underestimated.

So far, the only studies of DAMPs in humanized mice were focused on ATP. The first showed that the blockade of purinergic receptors (CD73 and CD39, which hydrolyze extracellular ATP to adenosine) resulted in aggravated GVHD [121]. Three others showed that blockade of P2X7, the main ATP-gated receptor on T cells, reduced GVHD severity [122–124]. DAMPs, here ATP, could therefore play a role in GVHD in humanized mice. However, none of these studies used TBI before transplanting NSG mice, and mice can be transplanted without TBI, meaning that conditioning-released DAMPs are not required for GVHD initiation. Interestingly, TBI was shown to increase the engraftment (frequency among human + mouse leukocytes) of human CD45^+^ cells in BM and spleen, without affecting the frequencies of CD3^+^, CD4^+^, CD8^+^, or CD19^+^ cells. This suggests that TBI enables the survival of lower amounts of human cells (vs. TBI-free protocols) through the elimination of residual mouse hematopoietic cells, further increasing the lymphopenia as well as the availability of hematopoietic factors [104]. Another explanation could be the increased availability of medullar niches after TBI to allow T-cell infiltration and subsequent interactions with host hematopoietic cells (currently the main candidates for T-cell xenogeneic stimulation).

The absence of LNs raises the interesting question of where host APCs stimulate donor T cells. The prevailing theory is that secondary lymphoid organs (specifically LNs, spleen, and PPs) are the main place of interaction between naive donor T cells and host APCs after allo-HCT [64, 125]. In the absence of LNs in NSG mice, the spleen is the candidate of choice as a T-cell priming site. Accordingly, a recent article using PET imaging of radiolabeled CD3 antibodies (injection of PBMCs i.p. without TBI) reported important signals in the spleen three days after transplantation [126]. This was associated with a significant enlargement of the spleen in this study and others observed high levels of activated T cells in the spleen seven days post-transplantation [104, 127]. Accordingly, the depletion of murine CD11c^+^ dendritic cells in the spleen of NSG mice by infusions of human CD4^─^ invariant NKT lymphocytes mitigated GVHD [128]. Furthermore, another team showed that the frequency of T cells in the spleen was relatively stable between days 7 and 27 post-transplantation while it gradually increased in BM and blood, suggesting that cells primarily home in the spleen and then migrate to peripheral organs [102]. However, the implication of the canonical lymphoid organs in GVHD initiation has been recently challenged. Indeed, while they show a survival advantage, mice splenectomized and deficient for LNs and PPs development (LN/PP/Sp^−/−^) still develop multi-organ GVHD in mouse-to-mouse allogeneic transplantation [129, 130]. Similarly, in humanized mice, splenectomy (which is assumed to remove most of the hematopoietic APCs) did not prevent the activation of T cells [104]. While this does not exclude the spleen as a key T-cell priming site, it suggests that it can also take place in other organs. Interestingly, in LN/PP/Sp^−/−^ mice, the BM served as the main alternative T-cell priming site [129]. Indeed, (i) the BM is an efficient T-cell priming site [131], (ii) humanized mice present elevated infiltrations of T cells in their BM, (iii) BM T cells present an activation/differentiation phenotype identical to those present in the spleen [95], and (iv) TBI increases the frequency of human T cells in the BM of humanized mice (from ~ 10% without TBI to ~ 60% with TBI) [92, 104]. Given the peculiar organic features of NSG mice, it is likely that T-cell priming occurs in alternative tissues, in particular the BM, but also the liver [132] or other inducible gut- or lung-associated lymphoid tissues [133].

### Xenogeneic T-cell activation

The capacity of mouse APCs to activate human T cells has been robustly demonstrated in multiple experimental systems. In vitro, a recent article showed that isolated human CD4^+^ or CD8^+^ T cells proliferated and secreted cytokines, in an MHC-dependent manner, when co-cultured with murine dendritic cells [134]. Interestingly, the T-cell proliferative responses in the presence of either xenogeneic or allogeneic DCs were equivalent, suggesting that both types of stimulation might depend on similar molecular mechanisms. The in vitro capacity of NSG DCs to stimulate isolated human T cells has been demonstrated in another report [104]. In vivo, the TCR_human_-MHC_mouse_ interaction has been notably demonstrated by the usage of NSG mice deficient for either MHC-I, -II, or both types of molecules [92, 135]. Specifically, NSG mice lacking MHC-II molecules presented a slightly better survival than conventional NSG while the deficiency of MHC-I molecules greatly improved their survival. The suppression of both MHC-I and -II molecule expression conferred the best survival advantage, with mice becoming virtually resistant to GVHD (90% survival at day 100, and absence of GVHD symptoms in 13/15 mice at day 125). A key conclusion drawn from these experiments was the maintenance of the MHC-I-CD8 and MHC-II-CD4 specificity across species. Indeed, the absence of MHC-I molecules resulted in a greater relative proliferation of CD4^+^ T cells while the absence of MHC-II resulted in greater frequencies of CD8^+^ T cells [135]. The absence of both MHC molecule types, while it did not fully prevent T-cell engraftment, resulted in similar proportions of CD4^+^ and CD8^+^ T cells in comparison to conventional NSG mice.

Mice and humans share virtually the same set of protein-coding genes. However, these genes are only 85% identical in terms of nucleotide sequence (vs. 99.9% identical between any two humans [136]), meaning that 15% of peptides presented by MHC molecules of NSG mice are possibly immunogenic to human T cells [137]. Therefore, assuming that xenogeneic reactions involve a process of molecular mimicry similar to allogeneic reactions, and considering that humans and mice are fully MHC mismatched, one could expect that the antigenicity of NSG APCs to human T cells is tremendously elevated. Consequently, a highly polyclonal expansion of human T-cell clones in NSG mice can reasonably be expected. In humans, GVHD is generally considered to be associated with the expansion of a limited number of dominant T-cell clones [35–40]. However, a recent report showed that the T-cell reaction characterizing GVHD in haploidentical HCT recipients involved the expansion of an elevated number of different clones, prompting the authors to characterize it as polyclonal [138]. They also evidenced that GVHD was linked to the important proliferation of low numbers of clones in the graft, in agreement with mathematical modeling of the T-cell population behavior when encountering alloantigens [139]. Thereby, defining the GVHD reaction as either oligoclonal or polyclonal is tedious because it requires establishing a clear threshold above which T-cell expansion should be deemed polyclonal, and no longer oligoclonal [140]. Nevertheless, we can assume that T-cell expansion in GVHD tends to be more oligoclonal than in allo-HCT recipients not developing GVHD [35–40] and that it involves the expansion of an elevated number of T-cell clones, dramatically re-organizing the TCR repertoire [40, 138].

**Fig. 2:**
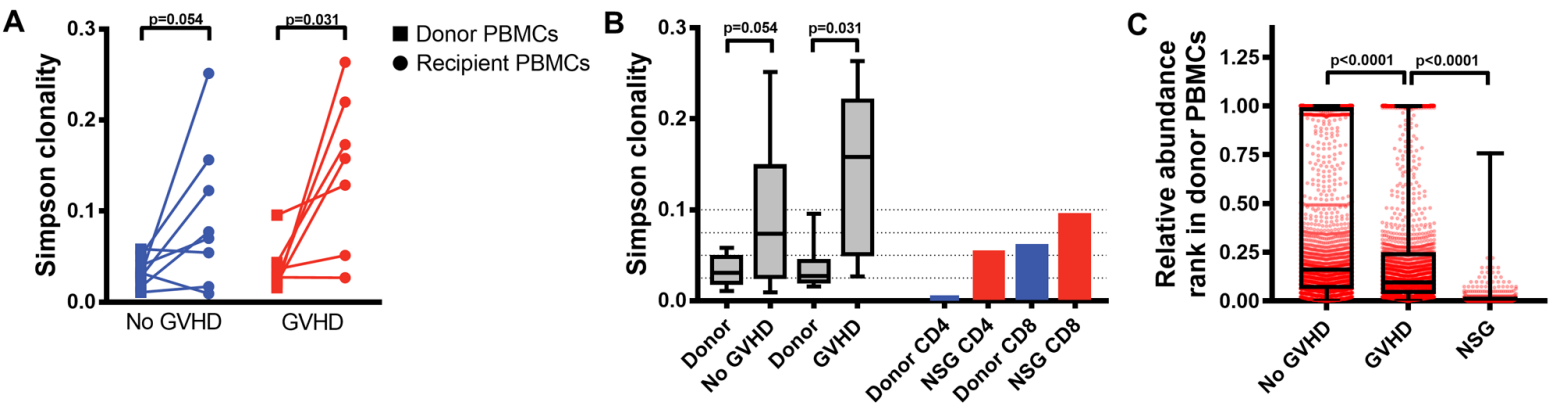
Comparison of T-cell clonal expansion between allo-HCT human recipients and humanized NSG mice. **(A)** Paired Wilcoxon comparison of the Simpson clonality (reflecting the TCR diversity, higher values mean lower diversity) between PBMCs collected from the peripheral blood of the donor before transplantation or from PBMCs of haploidentical transplantation recipients, 1-year post-transplantation (ImmuneACCESS: Kanakry-2016-JCIInsight). Comparisons were made for patients having developed symptoms of acute GVHD (any grade) or for those remaining free of GVHD symptoms. **(B)** Same data shown in (A) but presented as box plots (extending from first to third quartiles), in addition to single measures of the Simpson clonality indexes on sorted CD4^+^ and CD8^+^ T cells collected either from donor PBMCs or from ten spleens of NSG mice pooled together at day 14 post-transplantation (ImmuneACCESS: ehx-2024-ji). **(C)** T-cell clonotypes were ranked based on their abundance among donor PBMCs, ranks were normalized on the total number of clonotypes (1 = highest abundance and 0 = lowest abundance), and clonotypes commonly found in recipient and donor PBMCs and whose frequency was increased in recipients vs. donors (considered as allo/xeno-reactive) were plotted together

In NSG mice, the TCR repertoire diversity of splenic T cells, determined by spectratyping, was originally deemed polyclonal [94]. However, recent analyses by next-generation TCR sequencing showed that it is reduced (more oligoclonal) on day 14 post-transplantation in comparison to donor T cells [95]. In addition, there was a very low overlap between T-cell clonotypes found in donor PBMCs and those found in spleens, suggesting that the clonotypes expanding in NSG mice have a low abundance (below detection threshold) in donor PBMCs. To get a quantitative comparison of these observations with human GVHD, we re-analyzed these TCR sequencing data and confronted them with others collected in allo-HCT patients [141]. We first compared the TCR diversity index (Simpson clonality) between allo-HCT recipients having developed GVHD vs. GVHD-free patients (both groups at 1-year post-HCT) vs. NSG mice at day 14 (Fig. 2A-B). This showed that the clonal diversity tended to be reduced in all conditions compared to donor PBMCs. Clonality indexes in NSG mice were comparable to those found in humans (falling between the first and third quartiles of values found in humans). However, when comparing the change of abundance rank among allo- / xeno-reactive T-cell clones (here considered as all clones commonly found in recipients and donor PBMCs and whose frequency has increased in recipient vs. donor), we found that human GVHD was characterized by an important expansion of lowly abundant clones, in agreement with the previous observations [138]. In NSG mice, this phenomenon was even greater (Fig. 2C). Altogether, these observations suggest that the mechanisms ruling the antigenic stimulation of T cells in NSG mice are comparable to those taking place in humans.

In light of the oligoclonal T-cell expansion that characterizes GVHD in NSG mice, the identity of the clonotypes that are expanding can be called into question. A recent study has demonstrated the existence of two distinct types of TCR: those whose sequence results from the activity of the terminal deoxynucleotidyl transferase (TDT) and those whose sequence is independent of this enzyme [142]. TDT is the enzyme mediating the insertion of nucleotides in the TCR genes during V(D)J recombination and is known not to be expressed by neonatal thymocytes, which derive from fetal hematopoietic stem cells [143]. Therefore, the diversity of neonatal TCRs depends solely on V(D)J recombination while TDT-dependent ones depend on both V(D)J and TDT activity. Consequently, neonatal clonotypes were found to be shorter, were more shared between individuals, and constituted the entire TCR repertoire of cord blood. They also persisted throughout life and were associated with poor risks of inducing GVHD when present at a greater frequency in the graft [142]. Recently, we explored whether this could also be observed in NSG mice by comparing the TCR repertoire of two distinct donors before transplantation and after expansion in NSG mice [144]. While an expected sharing of ~ 2% of the TCR repertoire was found before transplantation, this sharing decreased to ~ 0.5% in the spleen of animals, suggesting that xenogeneic GVHD is mediated by more private TCRs (TDT-dependent), as observed in humans. These observations may also provide a basis for explaining the higher risks of GVHD when using older donors in haploidentical transplantations [145, 146].

While the vast re-organization of the TCR repertoire could be interpreted as evidence of antigen-restricted T-cell responses, their presence and role in xenogeneic GVHD remains to be firmly demonstrated. Interestingly, the expression of HLA-A02 molecules by NSG mice (NSG-HLA-A2/HHD mice, still expressing their murine MHC molecules) only mildly aggravates the GVHD (vs. conventional NSG mice) when they are transplanted with HLA-A02^+^ PBMCs^96^. In contrast, these mice presented a higher expansion and slightly better effector function of CD8^+^ T cells, but not higher tissue damage, suggesting that the presence of MHC-matched and antigen-specific reactions in NSG mice would only contribute to slightly ameliorating the T-cell activation, without impacting dramatically the model. In contrast, the transplantation of HLA-A02^─^ PBMCs to NSG-HLA-A2/HHD mice aggravated the GVHD to a greater extent, while T cells were identical to those in NSG mice, suggesting that GVHD in NSG mice depends on the number of “MHC-mismatches” between host and recipient, rather than on the pure genetic disparity between them. Similar conclusions were made regarding the magnitude of GVHD severity in allo-HCT patients [147]. Therefore, these observations suggest that the xenogeneic GVHD depends more on the MHC mismatching than on the presentation of immunogenic peptides, mimicking the biological setting of human GVHD.

In addition to the TCR-MHC interaction, T cells need co-stimulatory signals to trigger their proliferation as TCR signaling in the absence of co-stimulation results in anergy [148]. Co-stimulatory molecules fall mainly in two distinct superfamilies: immunoglobulin-like (including notably CD28, CTLA-4, CD80, CD86, and ICOS) and TNFR-like (including OX40, CD137, CD40, and CD27 among others). As in the vast majority of adaptive immune responses, the interaction between CD80/CD86 receptors of APCs and CD28 receptors of T cells is considered a pivotal co-stimulatory event in GVHD [149–151]. Accordingly, blocking this interaction with the CTLA-4-Ig fusion protein Abatacept (CTLA-4 binds to CD80 and CD86 with a greater affinity and avidity than CD28) reduces the incidence of GVHD in patients [152, 153]. Likewise, in NSG mice, CTLA-4-Ig treatment completely prevents the development of GVHD [94, 106]. This is notably possible because the murine CD86 molecules were shown to co-stimulate human T cells (through CD28) [154]. However, direct evidence is lacking to support the cross-species reactivity of other receptors. Nevertheless, the blockade of ICOS (expressed by T cells) through the usage of monoclonal antibodies also ameliorates greatly GVHD in humanized mice, suggesting a cross-species reactivity (possibly with murine ICOS-L) for this receptor as well [155]. Furthermore, the inhibition of PD-1, CD26, and CD38 (the two latter being co-stimulatory receptors not belonging to the superfamilies mentioned above) was also showed to impact the severity of GVHD in NSG mice [156–158], in agreement with observations made in conventional mouse models of allo-HCT or patients [159–161].

### T-cell proliferation

Following their activation, T cells need the support of cytokines to survive and expand. Only IL-2, IL-4, IL-7, and IL-15 (and not TNF-α, IFN-γ, IL-9, IL-13, IL-1, and IL-6) can sustain the survival of activated T cells [162]. Interestingly, these cytokines share two common features: their receptors contain the common gamma chain (γc, CD132), and they can all activate the phosphorylation of STAT5, a key player in inducing T-cell proliferation [163, 164]. In allo-HCT, the lymphopenia created by the conditioning typically results in elevated plasma levels of γc cytokines (especially IL-7 and IL-15 46, 165), which are no longer consumed by resting T cells. This abundance is sufficient to stimulate the proliferation of transplanted T cells, independently of antigenic stimulation, in a process called homeostatic peripheral expansion (HPE). Specifically, IL-7 is essential to support the proliferation of transplanted naive T-cells (not stimulated by their cognate antigen) [166] while IL-15 is needed for memory T-cell expansion [167]. Additionally, naive, but not memory, T-cells need to interact with self-MHC/peptide complexes for their HPE and this is associated with their differentiation into memory T cells, despite the absence of foreign antigenic stimulation [168]. While the nature of the MHC peptides involved in HPE remains unclear, they are likely low-affinity self-peptides because HPE is not impaired in germ-free lymphopenic recipients [169]. Therefore, the main distinctive characteristic of cells undergoing HPE is that activation markers such as CD69 and CD25 are not upregulated, thus allowing them to be phenotypically distinguished from antigen-activated T cells [170, 171].

In humanized NSG mice, no evidence supports the role of either IL-7 or IL-15 in T-cell proliferation. Recently, our group failed to identify human IL-15 in the serum of NSG mice on day 25 post-transplantation [127], and murine IL-15 has little effect on human cells [116]. We also observed that the serum of non-transplanted NSG mice failed to induce the phosphorylation of STAT5 in human T cells. This latter observation was surprising as murine IL-7 is known to cross-react with human IL-7 receptor [172]. However, other reports showed that the murine IL-7 is ~ 100-fold less potent than human IL-7 for supporting human T cell development [173, 174]. Furthermore, despite important lymphopenia, NSG mice may have low levels of IL-7 due to their mutations hampering the function of the key organs producing IL-7 (LNs, thymus, and BM [175–177]). Indeed, NSG mice lack LNs and have only a vestigial thymus due to the absence of interaction of developing thymocytes with thymic epithelial cells [178, 179]. Thereby, the expansion of T cells in NSG mice depends mainly on cytokines different from IL-7 and IL-15.

Following their activation by NSG APCs, human T cells in the spleen start expressing activation markers such as CD69 (~ 30%)^105^, CD25 (~ 40%)^159^, and HLA-DR (~ 40%)^128^. They also start secreting important amounts of IL-2, as evidenced by intracellular flow cytometry staining [103], RT-qPCR [127], and abundant presence in the serum of NSG mice post-transplantation [103, 127]. Currently, IL-2 is considered the main cytokine supporting the expansion of human T cells in NSG mice. This is supported by several lines of evidence: (i) CD4^+^ T cells transplanted alone mediate a more severe GVHD than CD8^+^ T cells (at equivalent cell doses) because the latter cells fail to expand properly in the absence of CD4^+^ T cells [94, 104]. This was later attributed to the incapacity of CD8^+^ T cells to produce important amounts of IL-2, in contrast with CD4^+^ T cells [104]. (ii) Cyclosporin-A (a calcineurin inhibitor) almost completely prevents the engraftment of human cells [94]. (iii) In vivo expression of human IL-2 in NSG mice (through hydrodynamic injections or transgenic expression) dramatically accelerates T-cell engraftment and GVHD [135, 180]. Interestingly, artificial IL-2 expression reduces the CD4/CD8 T-cell ratio while treatments reducing the signaling of IL-2 increase it [127, 181], highlighting the dependence of CD8^+^ T cells from IL-2 in this model. This is further supported by other results showing that CD8^+^ T cells transplanted alone mediate severe GVHD in NSG mice transgenic for human IL-2^108^. Nevertheless, and while it is tempting to speculate that IL-2 is the sole cytokine supporting the proliferation of T cells in NSG mice, the possible role or other candidates such as IL-4 and IL-21 (another γc cytokine secreted by activated T cells), should not be neglected. Indeed, IL-4 is also present in NSG mice serum (at low levels [104]), and blocking IL-21 signaling reduced T-cell frequencies and ameliorated GVHD in humanized NSG mice [182].

Because of the peculiar immunological settings of humanized NSG mice, the nature of the main driving force for T-cell expansion has been a frequent matter of debate. A popular hypothesis states that HPE participates largely in this expansion. Notably, a recent review discussed the possible role of two distinct types of HPE [183]. The first, termed “slow HPE”, would involve low-affinity TCR stimulation by self-antigens (presented by human APCs), IL-7 signaling, and no co-stimulation signaling. The second type, “fast HPE”, would involve the recognition of high-affinity TCR ligands (such as microbial peptides from commensal species presented by human APCs), and would preserve the organization of the TCR repertoire. As pointed out by this review, fast HPE is unlikely to occur in NSG mice because it is greatly reduced when recipient mice are housed in germ-free conditions [169]. Thereby, slow HPE would be the main homeostatic contributor to T-cell engraftment in NSG mice. This contribution has also been suggested by other reviews [184, 185], and a research article [104]. However, the mechanisms of T-cell expansion in NSG mice discussed above diverge largely from the features of slow HPE, namely: (i) the probably poor availability and contribution of IL-7; (ii) the important overexpression of activation markers (and pro-inflammatory cytokines) characterizing antigenic-stimulated T cells; (iii) the poor survival and negligible contribution of human APCs (the main cells able to present self-antigens to T cells); (iv) the large-scale reorganization of the TCR repertoire, better mimicking allogeneic reactions of GVHD than slow HPE-driven (driven by low-affinity TCR ligands) reconstitution of T-cells expected in GVHD-free patients; (v) the necessity of co-stimulation; (vi) the high impact of calcineurin inhibitors on T-cell engraftment, contrasting with the mild effects of these drugs on T-cell HPE [186]. Altogether, these observations suggest that T-cell engraftment in NSG mice is mainly mediated by xeno-antigenic stimulation of T cells rather than by HPE.

The most direct effect of T-cell proliferation is the increase of the T-cell frequency among leukocytes in NSG mice. With TBI and 2–5 × 10^6^ transplanted PBMCs, this frequency can reach values close to 100% in blood and spleen, and close to 50% in BM [92, 95, 104, 127]. Without TBI and 20 × 10^6^ transplanted PBMCs, it remains limited to ~ 80% in the spleen, ~ 60% in blood, and only 5–10% in BM [92, 103, 104]. In addition to increasing engraftment, TBI also aggravates GVHD. Therefore, it could be concluded that the abundance of circulating human T cells is determinant in the development and severity of GVHD. Indeed, multiple treatments shown to mitigate GVHD in NSG mice also reduced the frequency or absolute counts of human CD45^+^ cells^95, 103, 127, 181^. However, the implication of engraftment in determining GVHD severity has been questioned by a recent article comparing different cellular parameters between NSG mice developing mild vs. severe symptoms of GVHD after transplantation of equal doses of PBMCs from the same donor [105]. Surprisingly, the authors found that both groups of mice presented the same levels of human CD45^+^ cell abundance in their blood and spleen. In addition, other groups showed that mitigating GVHD can be done without decreasing the engraftment [128, 157, 187, 188]. Finally, we have shown that co-treating NSG mice with rapamycin and 5-azacytidine reduced the engraftment of human T cells in comparison to each treatment given alone, without further ameliorating GVHD, in comparison to each drug given alone [127]. We have also shown that GVHD could be aggravated (through the co-injection of Th17 cells) or ameliorated (through the co-infusion of regulatory T cells) without altering the engraftment of human cells [189, 190]. Altogether, these findings suggest that GVHD in NSG mice depends not only on the abundance of human cells but also on other parameters, such as the differentiation of T cells discussed in the following section.

### T-cell differentiation and migration

The T-cell population present in the PBMCs of healthy individuals is characterized by a heterogeneous distribution across different subsets including naive (TN, CD45RA^+^CD62L^+^CCR7^+^CD27^+^), effector (TEFF, CD45RA^─^CD62L^─^CCR7^─^CD27^─^), central memory (TCM, CD45RA^─^CD62L^+^CCR7^+^CD27^+^), and effector memory (TEM, CD45RA^─^CD62L^─^CCR7^─^CD27^+^) T cells. Following activation, T cells differentiate in a stepwise process from TN to TCM, and finally to TEM/TEFF which are characterized by the strongest pro-inflammatory and cytotoxic properties [191]. In adults aged under 60, TN and TCM represent the majority of T cells with frequencies of 30–40% for TN, and 15–50% for TCM [192, 193]. Upon infusion in NSG mice, TN and TCM (therefore, the majority of infused cells) migrate to the spleen. This is notably permitted by their expression of CD62L, an adhesion molecule enabling the homing to secondary lymphoid organs, as evidenced by a recent article showing that treating ex vivo PBMCs with progesterone for 6 h enabled the long-term maintenance of the T-cell CD62L expression, even upon antigenic stimulation [194]. NSG mice transplanted with these cells presented respectively greater and lower numbers of T cells in their spleen and lungs compared to NSG mice receiving unmanipulated PBMCs. Interestingly, progesterone did not maintain the expression of CCR7, suggesting that CD62L is the main molecule involved in the homing to the spleen.

**Fig. 3:**
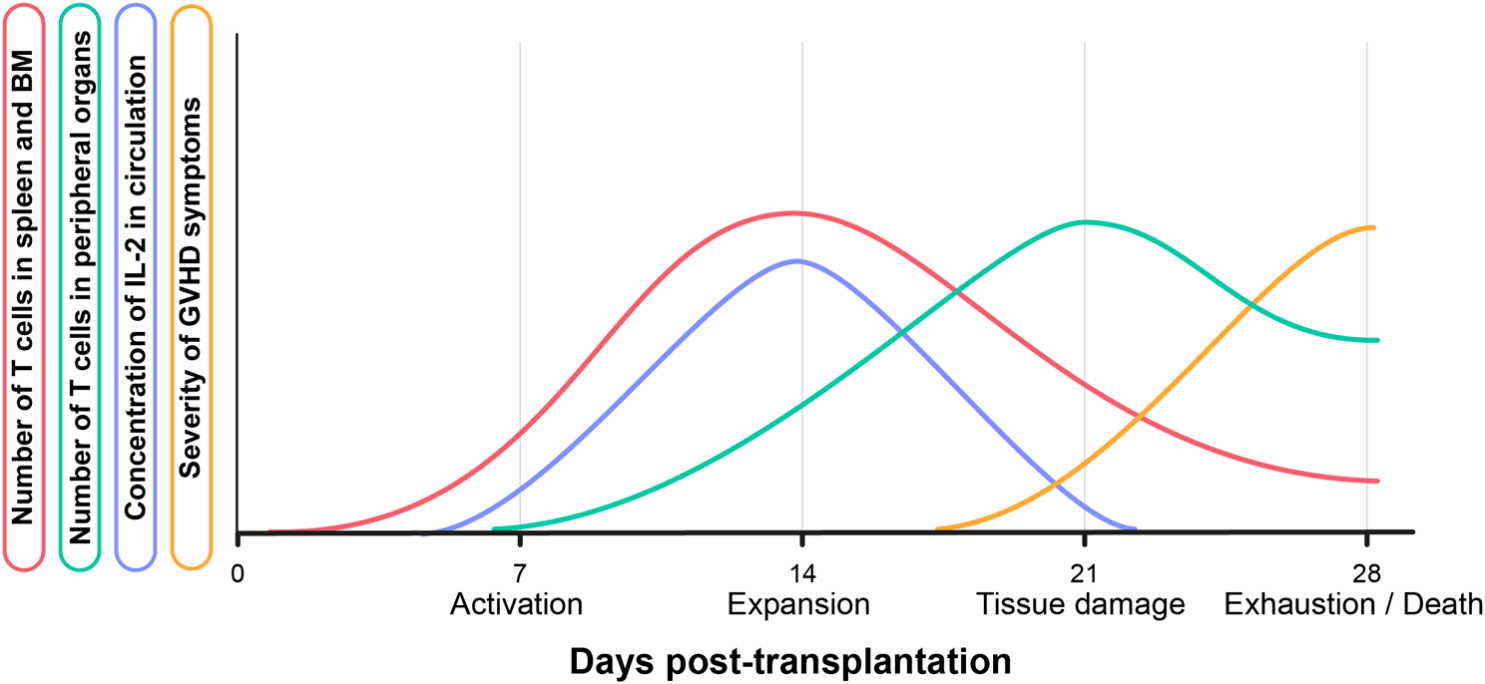
Theoretical dynamics of the evolution of T-cell numbers in the spleen, BM, and peripheral organs (liver and lungs mainly) as well as of the concentration of IL-2 in peripheral blood and severity of GVHD symptoms in NSG mice. The disease is divided into four phases over one month of experience, with the death of the animal occurring on day 28. These curves were theorized based on observations by our lab across multiple previous studies

Following their activation, TN and TCM differentiate into TEFF/TEM, able to leave the spleen and migrate toward peripheral organs (Fig. 3). This is notably supported by a report showing that transplanting sorted TN results after 7 days in a dominance of the TN/TCM phenotype in the spleen (~ 45% TN + ~ 19% TCM + ~ 36% TEFF/TEM), and a dominance of the TEFF/TEM phenotype in the lungs (~ 13% TN + ~ 34% TCM + ~ 54% TEFF/TEM) [104]. Accordingly, we showed that the liver and lungs present greater frequencies of TEFF than the spleen, BM, and blood on day 14 post-transplantation of PBMCs [95]. These were also the organs where we found the lowest frequencies of T cells secreting few pro-inflammatory cytokines. Importantly, due to the absence of thymic regeneration of the TN pool, the T-cell population only evolves toward a greater differentiation following transplantation, until being dominated by TEM cells (TN and TCM representing together only ~ 20% of T cells at day 14) [95].

In contrast with the TN/TCM, the transplanted TEFF/TEM do not express CD62L, and therefore they might home directly to peripheral organs after transplantation. While very little data are available about their fate, a prior study has observed that transfusing CD4^+^ T cells depleted from naive (CD45RA^+^) T cells resulted in dramatically delayed GVHD and engraftment [195]. It also reported that the depletion of TN resulted in “extremely rare” T cells in the spleen. In contrast, T cells were found in the liver, lung, skin, and colon, and the T-cell frequency was significantly lower than without TN depletion in multiple organs. Accordingly, other investigators showed that infusing only CCR7^+^ or CD62L^+^ T cells (mainly TN) accelerated the engraftment of CD4^+^ T cells [196]. Altogether, these observations are in concordance with the lower incidence of GVHD observed in patients given CD45RA-depleted grafts [197] as well as with observations in mouse-to-mouse models of GVHD [198]. These data indicate that transplanted TEM / TEFF (which are by definition T cells recognizing human or pathogen-derived antigens, not murine antigens) home to peripheral organs after transplantation and play a minor role in xenogenic GVHD pathophysiology.

In addition to the loss of CD62L, multiple other surface molecules participate in the tropism of TEFF/TEM toward (specific) peripheral organs. In allo-HCT, this tropism is mainly conducted by chemotaxis, and therefore by receptors able to bind chemokines released by tissues damaged by the conditioning. In humanized NSG mice, the possible participation of some of these receptors has been documented. However, these results are mainly correlative, and further investigations will be necessary to robustly demonstrate their role in T-cell homing or GVHD. Specifically, CLA (cutaneous lymphocyte antigen) and CCR4 were found to be more expressed by T cells infiltrating the skin than those present in the spleen [107]. Others showed that the CLA expression by circulating T cells is higher when mice start developing GVHD symptoms than on day 7 post-transplantation [102]. Another study showed that liver, lung, colon, and skin-infiltrating T cells express higher CXCR6 levels than peripheral T cells, suggesting that it is involved in the homing of T cells to GVHD target organs [195]. They also found higher expression of CCR9 by colon-infiltrating T cells vs. in the periphery.

In the course of their differentiation into TEFF, T cells acquire the expression of effector molecules that will determine their ultimate function. As in human GVHD, Th1 cells were found to be a prominent effector cell subset in xenogeneic GVHD. This was notably highlighted by the overexpression of specific gene sets in RNA sequencing of spleen T cells, IFN-γ / TNF-α secretion assays by flow cytometry (60–80% of secreting cells in the spleen, liver, and lungs at day 14), TBX21 expression by RT-qPCR, and elevated IFN-γ + TNF-α plasma levels by ELISA assays [94, 95, 127]. Interestingly, higher levels of serum IFN-γ were found in NSG mice having clinical vs. subclinical GVHD (with similar engraftment rates), highlighting the importance of Th1 differentiation in xGVHD pathogenesis [105]. However, the role of IFN-γ in xGVHD remains to be determined as another report showed that a P2X7 receptor antagonist reduced the serum IFN-γ concentration (~ 2-fold), without diminishing the engraftment and GVHD lethality (whereas it mildly reduced tissue damage) [123].

After Th1, Th17 is the second-best reported effector subset in xGVHD. The presence of Th17 has been evidenced by flow cytometry (IL-17 expression by CD4^+^ T cells, in spleen [103, 123], blood [128, 189], and at very low levels in the liver [95]), RT-qPCR of RORC [103, 127], and ELISA assays of IL-17 in serum. However, their frequency is typically low (1–3% of CD4^+^ T cells, lower than in transplanted PBMCs (5%)^96, 189^, and therefore the conditions in humanized NSG mice might be sub-optimal to support their adequate function and differentiation. Indeed, we failed to evidence a significant upregulation of a Th17 gene signature in the spleen of NSG mice (on day 14) compared to donor PBMCs [95]. Nevertheless, the participation of these cells in GVHD is relatively well-supported. First, the co-injection of Th17-polarized CD4^+^ T cells with PBMCs aggravated significantly the GVHD while the co-injection of non-polarized cells had no effect [189]. Second, upon injection of CD4^+^ T cells, NSG mice developed signs of skin inflammation (alopecia), and Th17s were found at greater levels in the skin than in the spleen (~ 2-fold) [107]; this alopecia developed faster when transplanting NSG mice transgenic for the Th17-promoting cytokines IL-1β and IL-23 199. Mechanistically, alopecia has been attributed to IL-17 as treating mice with anti-human IL-17 antibody (secukinumab) greatly ameliorated skin symptoms. In addition, there was a reduction of mouse neutrophil infiltration in the skin after secukinumab treatment, as well as prevention of alopecia by the removal of neutrophils with anti-Ly6G treatment, suggesting that alopecia results from the recruitment of murine neutrophils by IL-17. Third, mice with clinical GVHD have greater expression of IL-17 in their intestine (skin was not assessed) than mice with subclinical GVHD [105].

Altogether, these previous reports show that transplanted TN/TCM human T-cells primarily migrate to the spleen of the mice where they become activated and start differentiating into TEFF/TEM. They also acquire a Th1 or Th17 polarization. These cells then leave the spleen to migrate toward peripheral organs where their effector function will result in tissue damage.

### Tissue damage and exhaustion

**Fig. 4:**
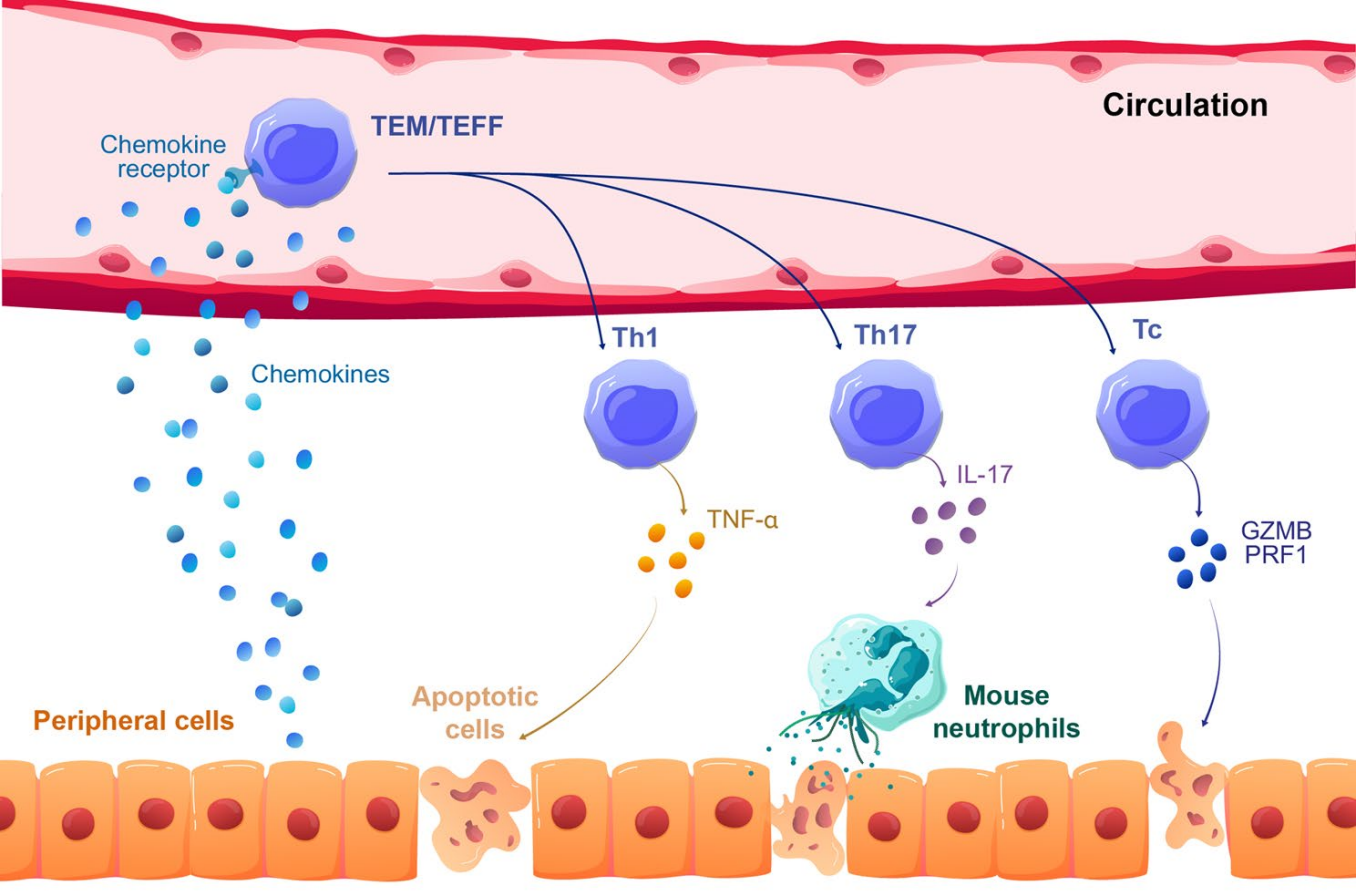
Illustration of the different pathways used by T cells to mediate tissue damage in NSG mice. Following the detection of specific chemokines secreted by the target organ, T cells cross the vasculature and infiltrate the organ where they attack healthy murine cells. Th1, T helper 1; Th17, T helper 17; Tc, T cytotoxic; GZMB, Granzyme B; PRF1, Perforin-1; TEM, Effector-memory T cells; TEFF, Effector T cells

Human T cells cause significant damage to the peripheral organs of NSG mice, with CD4^+^ and CD8^+^ T cells acting through different mechanisms (Fig. 4). CD4^+^ T cells secrete TNF-α, as observed by flow cytometry (~ 50% TNF-α^+^ cells in the spleen and liver, ~ 70% in the lungs) [95], and by ELISA assays on serum samples [95]. The role of TNF-α in xGVHD was highlighted by symptom relief following etanercept treatment, a TNF-α neutralizing agent [92, 94]. CD8^+^ T cells (30–40% of them) also secrete TNF-α in the spleen, liver, and lungs [95] and produce granzyme B and perforin-1 (~ 70% positive cells in the spleen), contributing to terminal tissue damage [103, 127]. Accordingly, depleting CD8^+^ T cells from the graft or transplanting CD4^+^ T cells alone reduces GVHD lethality and leads to chronic symptoms like alopecia [104, 107]. This suggests that CD4^+^ cells mainly support proliferation, while CD8^+^ T cells are responsible for tissue damage. Indeed, CD8^+^ T cells fail to expand in the absence of CD4^+^ T cells but when transplanted in mice transgenic for human IL-2, they proliferate robustly, causing severe acute GVHD and 100% mortality^108^.

Histologically, T-cell infiltration and tissue damage are observed in multiple organs, as discussed above. Notably, typical histologic signs of human GVHD (such as apoptotic bodies or bile plugs) can be found in the liver and lungs of NSG mice [95]. Furthermore, reflecting BM damage and reduced hematopoietic output, humanized NSG mice also present lower levels of hematocrit, red blood cells, platelets, and hemoglobin [92]. The development of anemia symptoms can also probably be attributed to the destruction of the BM. Finally, liver damage results in dramatically increased levels of alanine transaminase and aspartate transaminase in the plasma [94].

T-cell exhaustion occurs after repeated activation of T cells during chronic infection or tumor progression, but also after allo-HCT in response to alloantigen stimulation [200, 201]. The phenotype associated with exhaustion is defined by poor effector function, impaired proliferative capacity, and sustained expression of inhibitory receptors such as PD-1 and CTLA-4 [202]. These cells are also more prone to undergo apoptosis [203, 204]. In TBI-conditioned humanized NSG mice, many results suggest that T cells eventually reach an exhaustion state. First, the majority of T cells (~ 75%) express PD-1 and CTLA-4 in multiple organs [95, 106, 205]. Second, CD8^+^ T cells acquire the expression of CD4 molecules as a result of their chronic activation [205]. Third, splenic T cells have a low expression of BCL-2, and ~ 25% of them are apoptotic [127]. Fourth, treating NSG mice with anti-PD-1 antibodies promotes the T-cell eradication of lung cancer cells, showing that PD-1 expression by T cells results at least in part from exhaustion, and not only from activation (as PD-1 is also an activation marker) [206]. Fourth, when T-cells isolated from the spleen of irradiated mice (on day 25) were transfused to other naive NSG mice, these new animals failed to engraft and develop GVHD [127]. In contrast, when the same protocol was followed with T cells obtained from the spleen (on day 28) of non-irradiated humanized NSG mice, 100% of transplanted animals died from GVHD [103]. Altogether, these results indicate that T cells in irradiated mice (transplanted with 2 × 10^6^ PBMCs) enter an exhaustion state, possibly because of their chronic activation and/or because they need to undergo more cell divisions than their counterparts in non-irradiated mice (transplanted with 20 × 10^6^ PBMCs) to kill the mice. Nevertheless, it cannot be ruled out that T cells in non-irradiated mice also become exhausted after having undergone as many cell divisions as in irradiated mice. While more analyses will be needed to understand this phenomenon (which could also be replicative senescence), this suggests that TBI affects the long-term proliferative/functional capacity of transplanted T cells.

Due to the loss of T-cell effector / replicative capacity with time, it could be expected that some mice showing good engraftment rates survive the acute phase of the disease. Indeed, it is frequently observed that some animals survive in the long term, either because they have received GVHD-preventing therapies or because T cells failed to kill them [100, 103]. Then, as in humans, they tend to develop signs of chronic GVHD with liver/lungs/skin fibrosis, hair loss, alopecia, permanent weight loss, hunching, and sometimes eye keratinization (unpublished observation). In those mice, it could be hypothesized that CD8^+^ T cells became exhausted, leading to symptoms similar to those seen in mice transplanted with CD4^+^ T cells only. Thereby, the mechanism leading to fibrosis could also involve IL-17 and murine neutrophils, as discussed in previous sections.

## Functional relevance of xenoreactive T cells for the study of allogeneic reactions

Humanized NSG mice offer the advantage of enabling the study of GVHD mediated by human T cells in vivo. As detailed in the previous sections, T cells are the main mediator of the disease, and they share many features with those mediating GVHD in humans. However, the relatively high artificiality of the model legitimately raises the following question: do xenoreactive T cells have specific molecular features that distinguish them from T cells activated in the absence of any other stimuli than TCR and CD28 activation? If not, studying the effects of experimental drugs on the biology of T cells (activation, phenotype, proliferation, …) in NSG mice would be as relevant as performing in vitro assays on T cells stimulated with anti-CD3/CD28 antibodies. Previously, we performed RNA sequencing (RNA-seq) on T cells isolated from the spleen of NSG mice, seven days after transplantation, and we compared these cells with those before transplantation [95]. This comparison revealed the overexpression of multiple gene sets specific to activated T cells, namely TCR, CD28, mTOR and IL-2 signaling, proliferation pathways, and Th1/2/17 differentiation signatures. However, such pathways are also typically induced after activation by CD3/CD28 antibodies and are therefore not specific to xenoreactive T cells. Since we included T cells stimulated in vitro with CD3/CD28 antibodies as positive controls of activation in our RNA-seq analyses, we investigated hereafter the differences between T cells in the spleen of NSG mice and those activated in vitro.

**Fig. 5:**
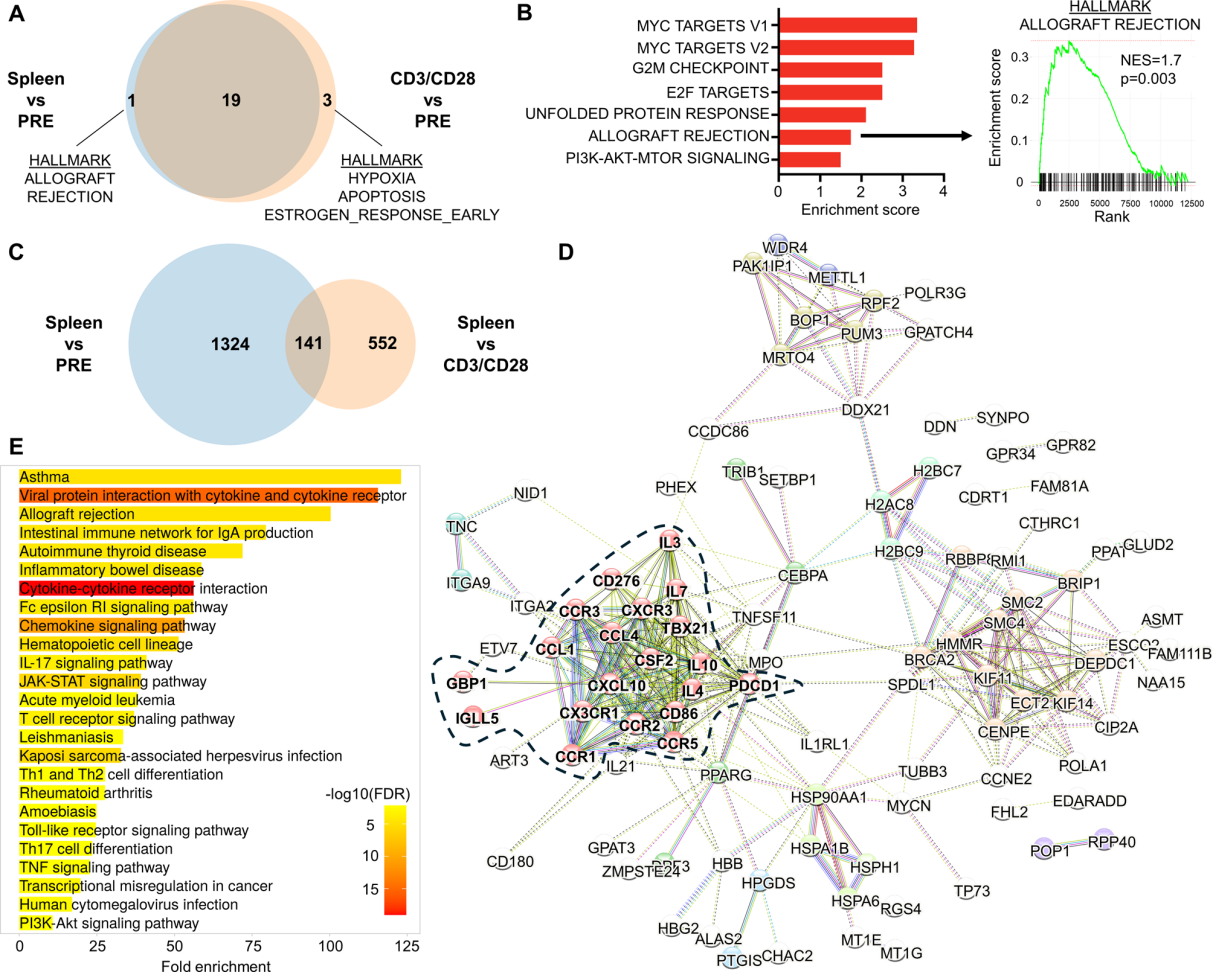
Xenoreactive T cells present specific molecular features of alloreactive T cells. RNA sequencing has been performed previously [95] (Arrayexpress: E-MTAB-6865) on T cells either before transplantation, isolated from the spleen of NSG mice on day seven post-transplantation, or stimulated with CD3/CD28 antibodies for four days in vitro. **(A)** Venn diagram comparing the HALLMARK gene sets that are significantly (*p* < 0.05) upregulated by splenic or CD3/CD28-stimulated T cells vs. pre-transplantation (PRE) T cells in GSEA analyses. **(B)** Enrichment scores of HALLMARK gene sets that are significantly upregulated by splenic vs. CD3/CD28-stimulated T cells in GSEA analyses. **(C)** Venn diagram comparing the identity of genes that are significantly (FDR < 0.05 and log_2_(fold-change) > 2) upregulated in indicated differential gene expression analyses (performed with limma-voom as described previously [275]). **(D)** STRING analysis has been performed with the online portal (https://string-db.org/) and default parameters on the 141 genes common to both analyses in panel C. DBSCAN analysis has been performed within the same portal and evidenced ten clusters, the main cluster (20 genes) is highlighted in red and manually circled with a dotted line. **(E)** ShinyGO analysis has been performed on the 20 genes of panel D with the online portal (http://bioinformatics.sdstate.edu/go/) and default parameters. NES, normalized enrichment score; FDR, false-discovery rate

Our previous analyses showed that hundreds of genes present a differential expression between NSG mice and in vitro-stimulated T cells [95]. However, we did not conduct any type of functional annotations on these genes, so it is unclear whether they represent specific biological functions or are simply noise resulting from differences in experimental conditions and/or time points post-activation. Here, we performed a gene set enrichment analysis (GSEA) on genes that are significantly upregulated by splenic (on day seven post-transplantation) and CD3/CD28-stimulated T cells (four days of stimulation) vs. those before the transplantation (PRE, Fig. 5A). As expected, the vast majority of gene sets were common to both conditions. However, one was specific to splenic T cells (allograft rejection), and three others, less relevant, were specific to CD3/CD28 T cells (hypoxia, apoptosis, and estrogen response). Furthermore, a direct comparison of splenic T cells vs. CD3/CD28-activated ones evidenced the significant upregulation of seven pathways by splenic cells, among which allograft rejection was also found (Fig. 5B). This shows that T cells expanding in NSG mice can be discriminated from in vitro-activated T cells based on molecular features that are specific to the allograft rejection process, supporting the relevance of xenoreactive T cells for the study of allogeneic reactions.

To provide more insights into this observation, we compared the identity of genes that are upregulated by splenic T cells vs. PRE-T cells (genes that are activated following xenogeneic stimulation) and those that are upregulated by splenic T cells vs. CD3/CD28 ones (genes that are specific to xenogeneic reactions in comparison to conventional activation). This provided a list of 141 genes (Fig. 5C). STRING and DBSCAN [207] clustering analysis on these genes highlighted the presence of a tightly interconnected cluster of 20 immune-related genes (Fig. 5D; Table 2). A ShinyGO analysis on these genes revealed significant functional associations with multiple pathways relevant to GVHD biology such as allograft rejection, chemokine signaling, Th17 differentiation, JAK-STAT signaling (notably involved in IL-2 signaling), TCR signaling, Th1/2 differentiation, and TNF signaling (Fig. 5E).

**Table 2:** List of the 20 genes composing the immunological cluster that is enriched in splenic T cells when compared to PRE or CD3/CD28-stimulated T cells. Indicated fold-changes and false-discovery rates (FDR) were obtained from the spleen vs. CD3/CD28 T cells differential gene expression analysis, performed with the limma-voom bioinformatic pipeline on previously published RNA-seq data [95]

Gene name	Symbol	Log_2_(fold-change)	FDR
Interleukin-10	IL10	7.61	7.5 × 10^− 5^
Interleukin-3	IL3	7.33	3.46 × 10^− 5^
C-X3-C Motif Chemokine Receptor 1	CX3CR1	5.93	1.36 × 10^− 5^
C-C chemokine receptor type 2	CCR2	5.80	0.00063
C-C chemokine receptor type 3	CCR3	5.59	0.00039
C-C chemokine receptor type 5	CCR5	5.31	0.0013
C-C chemokine receptor type 1	CCR1	5.26	0.00034
Chemokine (C-C motif) ligand 1	CCL1	4.45	0.0022
Interleukin-4	IL4	4.30	0.00015
C-X-C Motif Chemokine Ligand 10	CXCL10	4.20	0.012
Interleukin-7	IL7	3.82	0.0015
B7 Homolog 3 (B7-H3)	CD276	3.56	0.0023
Immunoglobulin lambda like polypeptide 5	IGLL5	3.08	0.043
Chemokine (C-C motif) ligand 4	CCL4	3.01	0.00071
Interferon-induced guanylate-binding protein 1	GBP1	2.96	9.05 × 10^− 5^
Programmed Cell Death 1 (PD-1)	PDCD1	2.54	0.0018
B7-2	CD86	2.25	0.011
Granulocyte-macrophage colony-stimulating factor (GM-CSF)	CSF2	2.11	0.001
C-X-C Motif Chemokine Receptor 3	CXCR3	2.07	0.00092
T-bet	TBX21	2.03	0.00023

Altogether, these observations show that xenogeneic T cells present multiple features that make them different from simply activated T cells. In particular, the specific expression of multiple chemokine-related genes highlights the relevance of the model to study the migration of T cells. The expression of CD276, CD86, and PD-1 supports its relevance to studying immune checkpoint pathways. Finally, the expression of multiple cytokines suggests that xenogeneic T cells generate a specific pro-inflammatory environment. Interestingly, among the 20 most specific immune-related genes (Table 2), the most upregulated one was IL-10. Previous reports suggested that abnormally high levels of IL-10 might play a role in clinical GVHD [208–210], and increasing its serum concentration in NSG mice dramatically accelerates GVHD [211]. While these data suggest that IL-10 plays a crucial role in xenogeneic GVHD, the exact mechanisms behind this role remain to be investigated.

In addition to the gene expression profile of xenogeneic T cells, other lines of evidence support the relevance of the model to study T-cell biology in GVHD. Specifically, two recent studies reported the possible role of CD4/CD8 double-positive T cells in clinical GVHD, and both studies observed the presence of this subset in NSG mice (whereas reaching opposite conclusions about their role in the disease) [205, 212]. In our experience, such T cells are typically not observed in vitro. Additionally, our team has demonstrated that T-cell phenotypic changes in response to a GVHD prophylactic regimen (Rapamycin) were identical in NSG mice and allo-HCT human recipients [127]. Moreover, another team evidenced remarkably similar serum concentrations of cytokines (34 tested, 10 significantly different but only 3 were dramatically different: IFN-γ, IL-10, and GM-CSF) between humanized NSG mice and their corresponding human donors [206]. Together with other similarities discussed herein (such as the TCR repertoire reorganization, the role of pro-inflammatory cytokines and effector T cells, the similarities between allo- and xeno-reactions, the tropism of T cells toward peripheral organs, and the role of immunomodulatory cells), these observations advocate in favor of the reliability of the humanized NSG mouse model to study GVHD.

## Humanized NSG mice: a platform to evaluate treatment response

While humanized NSG mice present some limitations to studying the biological mechanisms of human GVHD, we advocate that they are an excellent model to evaluate the response of T cells to diverse therapeutic options, either well-established or novel (such as aurora kinase A inhibitor [213], anti-CD26 antibodies [157], anti-CD45RC antibodies [214], Brilliant Blue G [215], miR-155 inhibition [216], Tocilizumab [217], and Abatacept [94]).

### Well-established GVHD prophylaxis and therapies

As could be expected, several conventional pharmacological agents used to treat GVHD in the clinical setting also ameliorate GVHD in humanized NSG mice. Specifically, the first-line systemic therapy for GVHD, methylprednisolone, showed an impressive efficacy at preventing GVHD with 100% of surviving treated animals at the time of death of the last control mouse [181]. Regarding prophylactic agents, Cyclosporin-A showed excellent responses, comparable to methylprednisolone [94, 181], while Tacrolimus showed a more mitigated response (but still ameliorated survival) [94]. Mycophenolate mofetil [181], post-transplant cyclophosphamide [144, 218–220], and ATG also ameliorate GVHD [219]. To our knowledge, methotrexate has never been assessed in humanized NSG mice so far. Importantly, these drugs also ameliorated GVHD in conventional mouse-to-mouse models of transplantation [221–224] (Table 1). Altogether, these previous studies evidence the expected response from GVHD to these conventional therapies.

### Regulatory T cells

**Fig. 6:**
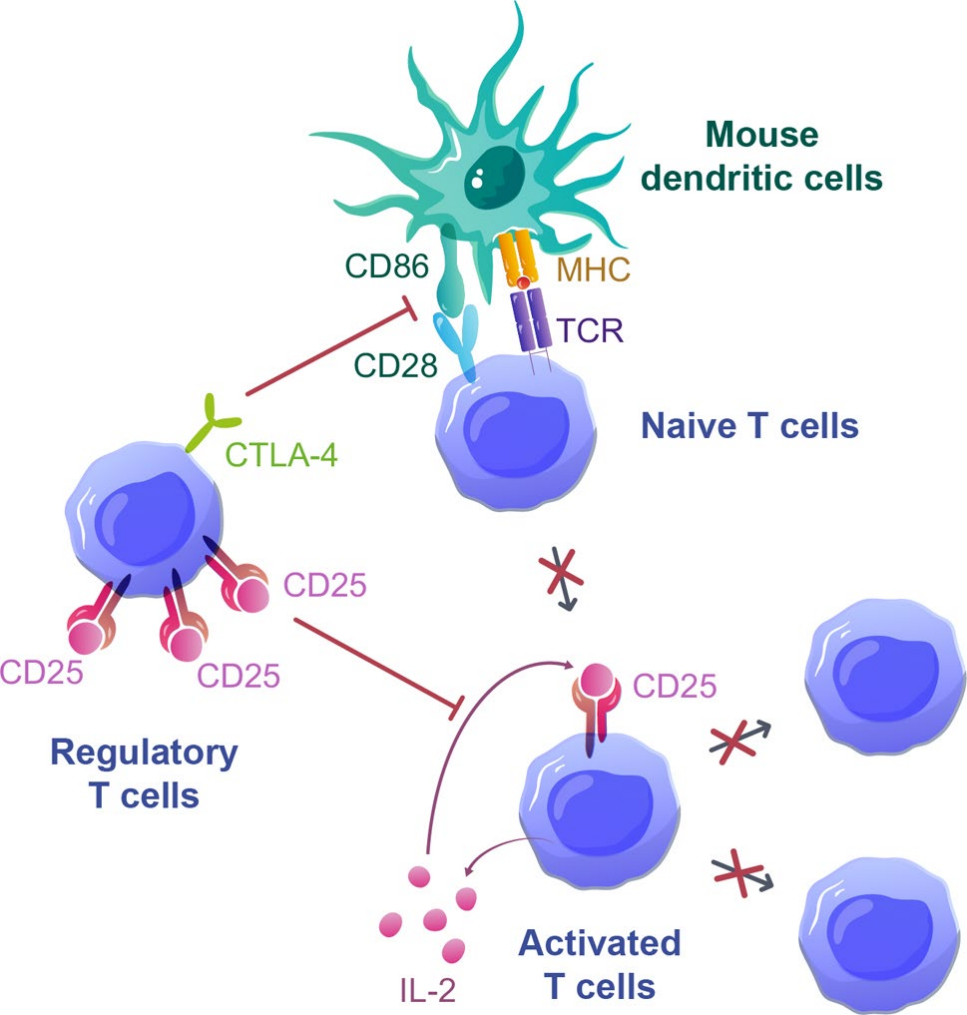
Main pathways used by Tregs to inhibit conventional T cells. The high expression of CTLA-4 by Tregs prevents the co-activation of conventional T cells by preventing the interaction between CD28 and CD86. The constitutively high expression of CD25 enables the capture of IL-2 by Tregs, depriving conventional T cells of this cytokine and preventing their proliferation

In addition to pharmacological agents, many immunoregulatory strategies aim at infusing or promoting the proliferation of cells able to mitigate GVHD [225]. Among these cells of interest, regulatory T cells (Treg) have been the focus of intense investigations. Tregs are CD4^+^ T cells able to suppress the effector function and proliferation of conventional T cells (both CD4^+^ and CD8^+^) [227]. This is notably possible through their elevated expression of CTLA-4 (which hampers the co-stimulation of conventional T cells by APCs) and their high consumption of IL-2, reducing the availability of this cytokine for the growth of other T cells (Fig. 6). Importantly, Tregs depend tightly on IL-2 (and not on IL-7) to sustain their function and proliferation [227]. This dependence is notably mediated by their constitutive high expression of CD25, the high-affinity receptor of IL-2, and their low expression of CD127, the receptor of IL-7 [228]. In turn, IL-2 (but not IL-7) induces robust phosphorylation of STAT5 in Tregs, which eventually stimulates their function and proliferation [229, 230]. Tregs are also characterized by the expression of the transcription factor FOXP3 which participates in the establishment of their immunoregulatory function [231]. Treg infusions were shown to mitigate GVHD in conventional mouse models [42, 43, 232–234], and the stimulation of their expansion by the administration of low doses of IL-2 tended to mitigate GVHD in patients [235–237].

In humanized NSG mice, Tregs (CD4^+^CD25^high^CD127^low^FOXP3^+^) have been detected at variable frequencies in multiple organs, including the spleen, blood, BM, lungs, and liver [95, 103, 127]. Their frequency is typically included between 2 and 6% of CD4^+^ T cells (frequencies close to those observed in patients in the first 100 days post-transplantation [127]), and they tend to be found at greater levels in the spleen and BM than in peripheral organs or blood. The protective function of Tregs in xGVHD is supported by multiple observations. First, Tregs in the spleen of NSG mice are capable of suppressing the proliferation of conventional T cells [103]. Second, the adoptive transfer of Tregs mitigates GVHD severity and lethality in NSG mice [181, 190, 238, 239], as well as reduces the overall inflammation and expansion of conventional T cells in mice organs [126]. Third, the transplantation of Treg-depleted PBMCs exacerbates GVHD [144]. Fourth, inhibiting the suppressive activity of Tregs aggravates GVHD [240]. Fifth, multiple Treg-promoting therapies (Rapamycin, 5-azacytidine, JAK inhibitors, PT-Cy, .) ameliorate GVHD in humanized NSG mice [103, 127, 213, 241]. Sixth, mice presenting long-term stabilization of peripheral Tregs by 5-azacytidine were protected from GVHD [103].

Given the pivotal role of IL-2 in supporting the proliferation of T cells in NSG mice, the presence of Tregs in their organs and the prevention of GVHD by these cells is not surprising. Furthermore, IL-2 is probably the key factor involved in the regulation of Treg levels in NSG mice, as suggested by several lines of evidence: (i) the frequency of Tregs fades over time in blood and spleen, probably paralleling the decrease of IL-2 availability as conventional T cells consume it and lose their IL-2 secretion capacity due to their progressive exhaustion [242]; (ii) high Treg frequency can be preserved by administering low doses of IL-2 or by inducing an artificial expression of IL-2 with hydrodynamic injections of IL-2-coding plasmids [180, 242]; (iii) selectively promoting the phosphorylation of STAT5 by IL-2 in Tregs results in an amelioration of GVHD symptoms [243]; (iv) treatments which promoted IL-2-STAT5 signaling also increased Treg frequencies [103, 127]. However, another study showed that TGF-β also plays a key role in Treg function/differentiation in NSG mice as the blockade of its production through GARP inhibition significantly reduces their function and abrogated their ability to mitigate GVHD [240]. Altogether, these previous studies demonstrate that Tregs are present and functional in humanized NSG mice and that these animals are a reliable platform for studying the immunomodulatory properties of Tregs.

### CAR-Tregs

An important limitation to polyclonal Treg therapies is that only a small fraction of these cells has the adequate TCR specificity to recognize alloantigens, suggesting that increased potency could be achieved by engineering antigen-specific Tregs [244]. A promising approach involves transducing Tregs to express chimeric antigen receptors (CARs) that recognize specific target antigens. This is achieved by using a single-chain variable fragment (scFv) fused to an intracellular T-cell signaling domain. The first CAR-Tregs, developed to reduce adverse immune responses in allotransplantation, were designed to target foreign major histocompatibility complex (MHC) antigens. MHC CAR-Tregs have demonstrated effectiveness in a xenogeneic mouse model of GVHD [245]. Subsequent studies of other CAR-Tregs targeted against OX40L [246], or HLA-A2 [247, 248] also used NSG mice transplanted with PBMCs to validate the therapeutic efficacy of their products.

### Mesenchymal stromal cells

Mesenchymal stromal cells (MSCs) are multipotent progenitors present in the BM which are capable of differentiating into various cells, such as adipocytes, chondrocytes, and osteoblasts [249]. MSCs have also been successfully isolated from several other tissues, including adipose tissue, umbilical cord, umbilical cord blood, and placenta [250, 251]. Similarly to Tregs, these cells harbor a wide range of immunosuppressive properties, reviewed previously [252]. Therefore, the capacity of these cells to prevent GVHD has been investigated extensively in clinical trials, with mixed results. Indeed, while some phase II trials reported that MSC infusions successfully reduced GVHD incidence [253–255], a meta-analysis failed to demonstrate a significant impact of MSCs on GVHD outcome [256].

Several previous studies have evaluated the ability of BM- or cord-blood MSC to prevent GVHD in humanized NSG mice [100, 257–265]. While some observed better survival after MSC infusions [263], several others failed to highlight a significant benefit of MSCs on GVHD [100, 259, 265]. Interestingly, similar mixed results were also obtained in conventional mouse-to-mouse models of transplantation with studies concluding about beneficial [266] or absent [267] effects of MSCs on GVHD. Further research will be necessary to understand the role of MSCs in GVHD, as well as to elucidate their immunotherapeutic potential in the prevention of this disease.

### Myeloid-derived suppressor cells

Similar to Tregs, myeloid-derived suppressor cells (MDSCs) can reduce T-cell activation and prevent effector T cells from damaging host tissues, thereby lessening GVHD severity. The mechanisms underlying this suppression have been reviewed previously [268]. In humanized mice, MDSC infusion mitigated GVHD by promoting Tregs and reducing pro-inflammatory cytokines [269, 270]. Additionally, another study demonstrated that injecting supernatant from MDSC cell cultures could also alleviate GVHD in humanized mice, suggesting that MDSCs primarily exert their effects through the secretion of immunosuppressive molecules [271]. These findings highlight the potential of MDSCs as a therapeutic tool for managing GVHD.

## Conclusions and future directions

Within over a decade, the usage of humanized NSG mice to investigate GVHD response to treatments has expanded dramatically. Nowadays, this model is an important component of the toolbox of investigators aiming at discovering or better understanding novel immunomodulatory therapies. In the present review, we have described the molecular and cellular mechanisms of GVHD in these animals and have highlighted multiple similarities between the pathophysiology of xenogeneic GVHD and human GVHD. In addition to some original observations obtained from the re-analysis of previously published results, the considerations reported herein support the relevance of the model for the study of allogeneic reactions mediated by T cells as well as to study the effects of various treatments on them.

Nevertheless, the model has several limitations that will need to be addressed in the future to reach a model mimicking as closely as possible human allo-HCT. First, the model is based on xeno- instead of alloreactivity. This limitation could be circumvented by developing immunodeficient mice knock-out for murine MHC and transgenic for human MHC molecules. So far, such development is mainly exemplified by HUMAMICE (immunodeficient C57BL6 mice knock-out for mouse MHC and transgenic for HLA-A2 and HLA-DR1) [97]. Second, the absence of some key cytokines not or lowly secreted by T cells and playing pivotal roles in GVHD, such as IL-7 and IL-15. NSG mice transgenic for these molecules have notably been developed [272] and the investigation of GVHD and T-cell homeostasis in these animals is certainly warranted. Third, the hampered development of non-T cells post-transplantation. Again, such a limitation should be circumvented by the development of NSG mice expressing human cytokines such as the NSG-SGM3 (co-expressing IL-3, GM-CSF, and SCF) [273], or the above-mentioned IL-7/-15 double knockin NSG mice which show better engraftment of NK cells. Fourth, the low/absent infiltration in skin/intestines and the consequent absence of GVHD symptoms related to these organs. Interestingly, a recent article showed that pre-treating T cells with IL-7 before infusion in NSG mice dramatically increased their homing to the intestinal mucosa [274]. Therefore, NSG mice transgenic for human cytokines could show migration distributions toward peripheral organs better mimicking those found in humans. Fifth, the absence of LNs (which contributes to the absence of intestinal GVHD). This last limitation could be resolved through IL2rγ chain expression restricted to the lymphoid tissues.

Despite their limitations, humanized NSG mice provide valuable insights into the best approach to mitigate GVHD. They can also indirectly help to better understand the biological mechanisms ruling alloreactivity, T-cell activation, expansion, and migration to peripheral organs.

## Data Availability

The RNA-seq and TCR-seq data related to NSG mice have been published previously [95] and can be accessed on ImmuneACCESS (ehx-2024-ji) and Arrayexpress (E-MTAB-6865). The human TCR-seq data have been published previously [141] and can be accessed on ImmuneACCESS (Kanakry-2016-JCIInsight).

## References

[1] Shouval R, et al. Outcomes of allogeneic haematopoietic stem cell transplantation from HLA-matched and alternative donors: a European Society for blood and marrow transplantation registry retrospective analysis. Lancet Haematol. 2019;6:e573–84. 31477550 10.1016/S2352-3026(19)30158-9

[2] Zeiser R, Blazar BR. Acute Graft-versus-host disease - biologic process, Prevention, and Therapy. N Engl J Med. 2017;377:2167–79. 29171820 10.1056/NEJMra1609337 PMC6034180

[3] Baron F, et al. Graft-versus-tumor effects after allogeneic hematopoietic cell transplantation with nonmyeloablative conditioning. J Clin Oncol. 2005;23:1993–2003. 15774790 10.1200/JCO.2005.08.136

[4] Petersdorf EW. Genetics of graft-versus-host disease: the major histocompatibility complex. Blood Rev. 2013;27:1–12. 23182478 10.1016/j.blre.2012.10.001 PMC3626414

[5] Horowitz MM, et al. Graft-versus-leukemia reactions after bone marrow transplantation. Blood. 1990;75:555–62. 2297567

[6] Baron F. Abatacept vs PT-Cy for GVHD prophylaxis. Blood. 2024;144:1762–4. 39446374 10.1182/blood.2024026116

[7] Zeiser R, et al. Ruxolitinib for glucocorticoid-refractory Acute graft-versus-host disease. N Engl J Med. 2020;382:1800–10. 32320566 10.1056/NEJMoa1917635

[8] Servais S, et al. Novel approaches for preventing acute graft-versus-host disease after allogeneic hematopoietic stem cell transplantation. Expert Opin Investig Drugs. 2016;25:957–72. 27110922 10.1080/13543784.2016.1182498

[9] Luznik L, et al. Randomized Phase III BMT CTN trial of calcineurin inhibitor-free chronic graft-versus-host Disease interventions in Myeloablative hematopoietic cell transplantation for hematologic malignancies. J Clin Oncol. 2022;40:356–68. 34855460 10.1200/JCO.21.02293 PMC8797487

[10] Zhang Y, Louboutin JP, Zhu J, Rivera AJ, Emerson SG. Preterminal host dendritic cells in irradiated mice prime CD8 + T cell-mediated acute graft-versus-host disease. J Clin Invest. 2002;109:1335–44. 12021249 10.1172/JCI14989 PMC150980

[11] Hill GR, et al. Total body irradiation and acute graft-versus-host disease: the role of gastrointestinal damage and inflammatory cytokines. Blood. 1997;90:3204–13. 9376604

[12] Staffas A, Burgos da Silva M, van den Brink MR. The intestinal microbiota in allogeneic hematopoietic cell transplant and graft-versus-host disease. Blood (2016). 10.1182/blood-2016-09-691394 PMC5324712 27940475

[13] Zeiser R, Socie G, Blazar BR. Pathogenesis of acute graft-versus-host disease: from intestinal microbiota alterations to donor T cell activation. Br J Haematol. 2016;175:191–207. 27619472 10.1111/bjh.14295

[14] Stenger EO, Turnquist HR, Mapara MY, Thomson AW. Dendritic cells and regulation of graft-versus-host disease and graft-versus-leukemia activity. Blood. 2012;119:5088–103. 22403259 10.1182/blood-2011-11-364091 PMC3369606

[15] Kolb HJ, Schmidt C, Barrett AJ, Schendel DJ. Graft-versus-leukemia reactions in allogeneic chimeras. Blood. 2004;103:767–76. 12958064 10.1182/blood-2003-02-0342

[16] Shlomchik WD, et al. Prevention of graft versus host disease by inactivation of host antigen-presenting cells. Science. 1999;285:412–5. 10411505 10.1126/science.285.5426.412

[17] Koyama M, Hill GR. Alloantigen presentation and graft-versus-host disease: fuel for the fire. Blood. 2016;127:2963–70. 27030390 10.1182/blood-2016-02-697250

[18] Wang X, et al. Mechanisms of antigen presentation to T cells in murine graft-versus-host disease: cross-presentation and the appearance of cross-presentation. Blood. 2011;118:6426–37. 21963602 10.1182/blood-2011-06-358747 PMC3236124

[19] Colf LA, et al. How a single T cell receptor recognizes both self and foreign MHC. Cell. 2007;129:135–46. 17418792 10.1016/j.cell.2007.01.048

[20] Speir JA, et al. Structural basis of 2 C TCR allorecognition of H-2Ld peptide complexes. Immunity. 1998;8:553–62. 9620676 10.1016/s1074-7613(00)80560-9

[21] Reiser JB, et al. Crystal structure of a T cell receptor bound to an allogeneic MHC molecule. Nat Immunol. 2000;1:291–7. 11017099 10.1038/79728

[22] Macdonald WA, et al. T cell allorecognition via molecular mimicry. Immunity. 2009;31:897–908. 20064448 10.1016/j.immuni.2009.09.025

[23] Archbold JK, et al. Alloreactivity between disparate cognate and allogeneic pMHC-I complexes is the result of highly focused, peptide-dependent structural mimicry. J Biol Chem. 2006;281:34324–32. 16963442 10.1074/jbc.M606755200

[24] Morice A, et al. Cross-reactivity of herpesvirus-specific CD8 T cell lines toward allogeneic class I MHC molecules. PLoS ONE. 2010;5:e12120. 20711433 10.1371/journal.pone.0012120 PMC2920819

[25] Amir AL, et al. Allo-HLA reactivity of virus-specific memory T cells is common. Blood. 2010;115:3146–57. 20160165 10.1182/blood-2009-07-234906

[26] Gras S, Kjer-Nielsen L, Chen Z, Rossjohn J, McCluskey J. The structural bases of direct T-cell allorecognition: implications for T-cell-mediated transplant rejection. Immunol Cell Biol. 2011;89:388–95. 21301478 10.1038/icb.2010.150

[27] Smith C, Miles JJ, Khanna R. Advances in direct T-cell alloreactivity: function, avidity, biophysics and structure. Am J Transpl. 2012;12:15–26. 10.1111/j.1600-6143.2011.03863.x 22152064

[28] Sherman LA, Chattopadhyay S. The molecular basis of allorecognition. Annu Rev Immunol. 1993;11:385–402. 8476567 10.1146/annurev.iy.11.040193.002125

[29] Suchin EJ, et al. Quantifying the frequency of alloreactive T cells in vivo: new answers to an old question. J Immunol. 2001;166:973–81. 11145675 10.4049/jimmunol.166.2.973

[30] Wang Y, et al. How an alloreactive T-cell receptor achieves peptide and MHC specificity. Proc Natl Acad Sci U S A. 2017;114:E4792–801. 28572406 10.1073/pnas.1700459114 PMC5474831

[31] Eyrich M, et al. Distinct contributions of CD4(+) and CD8(+) naive and memory T-cell subsets to overall T-cell-receptor repertoire complexity following transplantation of T-cell-depleted CD34-selected hematopoietic progenitor cells from unrelated donors. Blood. 2002;100:1915–8. 12176918 10.1182/blood-2001-11-0005

[32] Arstila TP, et al. A direct estimate of the human alphabeta T cell receptor diversity. Science. 1999;286:958–61. 10542151 10.1126/science.286.5441.958

[33] Roux E, et al. Recovery of immune reactivity after T-cell-depleted bone marrow transplantation depends on thymic activity. Blood. 2000;96:2299–303. 10979980

[34] Haynes BF, Markert ML, Sempowski GD, Patel DD, Hale LP. The role of the thymus in immune reconstitution in aging, bone marrow transplantation, and HIV-1 infection. Annu Rev Immunol. 2000;18:529–60. 10837068 10.1146/annurev.immunol.18.1.529

[35] Yew PY, et al. Quantitative characterization of T-cell repertoire in allogeneic hematopoietic stem cell transplant recipients. Bone Marrow Transpl. 2015;50:1227–34. 10.1038/bmt.2015.133 PMC4559843 26052909

[36] Liu X, Chesnokova V, Forman SJ, Diamond DJ. Molecular analysis of T-cell receptor repertoire in bone marrow transplant recipients: evidence for oligoclonal T-cell expansion in graft-versus-host disease lesions. Blood. 1996;87:3032–44. 8639927

[37] Alachkar H, Nakamura Y. Deep-sequencing of the T-cell receptor repertoire in patients with haplo-cord and matched-donor transplants. Chimerism. 2015;6:47–9. 26745665 10.1080/19381956.2015.1128624 PMC5154355

[38] Liu C, He M, Rooney B, Kepler TB, Chao NJ. Longitudinal analysis of T-cell receptor variable beta chain repertoire in patients with acute graft-versus-host disease after allogeneic stem cell transplantation. Biol Blood Marrow Transpl. 2006;12:335–45. 10.1016/j.bbmt.2005.09.019 16503503

[39] Gkazi AS, et al. Clinical T cell receptor repertoire deep sequencing and analysis: an application to monitor Immune reconstitution following cord blood transplantation. Front Immunol. 2018;9:2547. 30455696 10.3389/fimmu.2018.02547 PMC6231291

[40] Leick M, et al. T cell Clonal Dynamics determined by high-resolution TCR-β sequencing in recipients after allogeneic hematopoietic cell transplantation. Biol Blood Marrow Transpl. 2020;26:1567–74. 10.1016/j.bbmt.2020.04.026 PMC8279830 32417490

[41] Brownlie RJ, Zamoyska R. T cell receptor signalling networks: branched, diversified and bounded. Nat Rev Immunol. 2013;13:257–69. 23524462 10.1038/nri3403

[42] Edinger M, et al. CD4 + CD25 + regulatory T cells preserve graft-versus-tumor activity while inhibiting graft-versus-host disease after bone marrow transplantation. Nat Med. 2003;9:1144–50. 12925844 10.1038/nm915

[43] Cohen JL, Trenado A, Vasey D, Klatzmann D, Salomon BL. CD4(+)CD25(+) immunoregulatory T cells: new therapeutics for graft-versus-host disease. J Exp Med. 2002;196:401–6. 12163568 10.1084/jem.20020090 PMC2193933

[44] Matsuoka K, et al. Low-dose interleukin-2 therapy restores regulatory T cell homeostasis in patients with chronic graft-versus-host disease. Sci Transl Med. 2013;5:179ra143. 10.1126/scitranslmed.3005265 PMC3686517 23552371

[45] Thiant S, et al. Plasma levels of IL-7 and IL-15 in the first month after myeloablative BMT are predictive biomarkers of both acute GVHD and relapse. Bone Marrow Transpl. 2010;45:1546–52. 10.1038/bmt.2010.13 20190846

[46] Thiant S, et al. Plasma levels of IL-7 and IL-15 after reduced intensity conditioned allo-SCT and relationship to acute GVHD. Bone Marrow Transpl. 2011;46:1374–81. 10.1038/bmt.2010.300 21132028

[47] Dean RM, et al. Association of serum interleukin-7 levels with the development of acute graft-versus-host disease. J Clin Oncol. 2008;26:5735–41. 19001329 10.1200/JCO.2008.17.1314 PMC2645098

[48] Piper C, et al. Single-cell immune profiling reveals a developmentally distinct CD4 + GM-CSF + T-cell lineage that induces GI tract GVHD. Blood Adv. 2022;6:2791–804. 35015822 10.1182/bloodadvances.2021006084 PMC9092418

[49] Perales MA, et al. Recombinant human interleukin-7 (CYT107) promotes T-cell recovery after allogeneic stem cell transplantation. Blood. 2012;120:4882–91. 23012326 10.1182/blood-2012-06-437236 PMC3520625

[50] Jiang H, Fu D, Bidgoli A, Paczesny ST. Cell subsets in Graft Versus host Disease and Graft Versus Tumor. Front Immunol. 2021;12:761448. 34675938 10.3389/fimmu.2021.761448 PMC8525316

[51] Burman AC, et al. IFNgamma differentially controls the development of idiopathic pneumonia syndrome and GVHD of the gastrointestinal tract. Blood. 2007;110:1064–72. 17449800 10.1182/blood-2006-12-063982

[52] Hill GR, Ferrara JL. The primacy of the gastrointestinal tract as a target organ of acute graft-versus-host disease: rationale for the use of cytokine shields in allogeneic bone marrow transplantation. Blood. 2000;95:2754–9. 10779417

[53] Robb RJ, Hill GR. The interferon-dependent orchestration of innate and adaptive immunity after transplantation. Blood. 2012;119:5351–8. 22517908 10.1182/blood-2012-02-368076

[54] Varelias A, et al. Acute graft-versus-host disease is regulated by an IL-17-sensitive microbiome. Blood. 2017;129:2172–85. 28137828 10.1182/blood-2016-08-732628 PMC5391622

[55] Cai Y, et al. Adoptively transferred donor IL-17-producing CD4(+) T cells augment, but IL-17 alleviates, acute graft-versus-host disease. Cell Mol Immunol. 2018;15:233–45. 27748733 10.1038/cmi.2016.37 PMC5843609

[56] Ratajczak P, et al. Th17/Treg ratio in human graft-versus-host disease. Blood. 2010;116:1165–71. 20484086 10.1182/blood-2009-12-255810 PMC3001485

[57] Park H, et al. A distinct lineage of CD4 T cells regulates tissue inflammation by producing interleukin 17. Nat Immunol. 2005;6:1133–41. 16200068 10.1038/ni1261 PMC1618871

[58] Hill GR, et al. Stem cell mobilization with G-CSF induces type 17 differentiation and promotes scleroderma. Blood. 2010;116:819–28. 20435882 10.1182/blood-2009-11-256495

[59] Forcade E et al. An activated Th17-prone T cell subset involved in chronic graft-versus-host disease sensitive to pharmacological inhibition. JCI Insight 2 (2017). 10.1172/jci.insight.92111 PMC5470889 28614794

[60] Yi T, et al. Reciprocal differentiation and tissue-specific pathogenesis of Th1, Th2, and Th17 cells in graft-versus-host disease. Blood. 2009;114:3101–12. 19602708 10.1182/blood-2009-05-219402 PMC2756212

[61] Foley JE, Mariotti J, Ryan K, Eckhaus M, Fowler DH. Th2 cell therapy of established acute graft-versus-host disease requires IL-4 and IL-10 and is abrogated by IL-2 or host-type antigen-presenting cells. Biol Blood Marrow Transpl. 2008;14:959–72. 10.1016/j.bbmt.2008.06.007 PMC2835696 18721759

[62] Furlan SN, et al. Systems analysis uncovers inflammatory Th/Tc17-driven modules during acute GVHD in monkey and human T cells. Blood. 2016;128:2568–79. 27758873 10.1182/blood-2016-07-726547 PMC5123196

[63] Gartlan KH, et al. Tc17 cells are a proinflammatory, plastic lineage of pathogenic CD8 + T cells that induce GVHD without antileukemic effects. Blood. 2015;126:1609–20. 26206951 10.1182/blood-2015-01-622662

[64] Panoskaltsis-Mortari A, et al. In vivo imaging of graft-versus-host-disease in mice. Blood. 2004;103:3590–8. 14715632 10.1182/blood-2003-08-2827

[65] Wysocki CA, Panoskaltsis-Mortari A, Blazar BR, Serody JS. Leukocyte migration and graft-versus-host disease. Blood. 2005;105:4191–9. 15701715 10.1182/blood-2004-12-4726 PMC1895033

[66] Piper KP, et al. CXCL10-CXCR3 interactions play an important role in the pathogenesis of acute graft-versus-host disease in the skin following allogeneic stem-cell transplantation. Blood. 2007;110:3827–32. 17766680 10.1182/blood-2006-12-061408

[67] Duffner U, et al. Role of CXCR3-induced donor T-cell migration in acute GVHD. Exp Hematol. 2003;31:897–902. 14550805 10.1016/s0301-472x(03)00198-x

[68] Terwey TH, et al. CCR2 is required for CD8-induced graft-versus-host disease. Blood. 2005;106:3322–30. 16037386 10.1182/blood-2005-05-1860 PMC1895329

[69] Rao AR, et al. CC chemokine receptor 2 expression in donor cells serves an essential role in graft-versus-host-disease. J Immunol. 2003;171:4875–85. 14568968 10.4049/jimmunol.171.9.4875

[70] Palmer LA, et al. Chemokine receptor CCR5 mediates alloimmune responses in graft-versus-host disease. Biol Blood Marrow Transpl. 2010;16:311–9. 10.1016/j.bbmt.2009.12.002 PMC3182111 20025985

[71] Murai M, et al. Active participation of CCR5(+)CD8(+) T lymphocytes in the pathogenesis of liver injury in graft-versus-host disease. J Clin Invest. 1999;104:49–57. 10393698 10.1172/JCI6642 PMC408408

[72] Varona R, Cadenas V, Gomez L, Martinez AC, Marquez G. CCR6 regulates CD4 + T-cell-mediated acute graft-versus-host disease responses. Blood. 2005;106:18–26. 15774622 10.1182/blood-2004-08-2996

[73] Varona R, et al. CCR6 regulates the function of alloreactive and regulatory CD4 + T cells during acute graft-versus-host disease. Leuk Lymphoma. 2006;47:1469–76. 16966255 10.1080/10428190500513819

[74] Tsuchiyama J, et al. Cutaneous lymphocyte antigen-positive T cells may predict the development of acute GVHD: alterations and differences of CLA + T- and NK-cell fractions. Bone Marrow Transpl. 2009;43:863–73. 10.1038/bmt.2008.392 19043457

[75] Petrovic A, et al. LPAM (alpha 4 beta 7 integrin) is an important homing integrin on alloreactive T cells in the development of intestinal graft-versus-host disease. Blood. 2004;103:1542–7. 14563643 10.1182/blood-2003-03-0957

[76] Du W, Cao X. Cytotoxic pathways in allogeneic hematopoietic cell transplantation. Front Immunol. 2018;9:2979. 30631325 10.3389/fimmu.2018.02979 PMC6315278

[77] Spoerl S, et al. Activity of therapeutic JAK 1/2 blockade in graft-versus-host disease. Blood. 2014;123:3832–42. 24711661 10.1182/blood-2013-12-543736

[78] Mestas J, Hughes CC. Of mice and not men: differences between mouse and human immunology. J Immunol. 2004;172:2731–8. 14978070 10.4049/jimmunol.172.5.2731

[79] Socié G, Kean LS, Zeiser R, Blazar BR. Insights from integrating clinical and preclinical studies advance understanding of graft-versus-host disease. J Clin Invest 131 (2021). 10.1172/JCI149296 PMC8203454 34101618

[80] Zeiser R, Blazar BR. Preclinical models of acute and chronic graft-versus-host disease: how predictive are they for a successful clinical translation? Blood. 2016;127:3117–26. 26994149 10.1182/blood-2016-02-699082 PMC4920018

[81] Bosma GC, Custer RP, Bosma MJ. A severe combined immunodeficiency mutation in the mouse. Nature. 1983;301:527–30. 6823332 10.1038/301527a0

[82] Mosier DE, Gulizia RJ, Baird SM, Wilson DB. Transfer of a functional human immune system to mice with severe combined immunodeficiency. Nature. 1988;335:256–9. 2970594 10.1038/335256a0

[83] Lapidot T, et al. Cytokine stimulation of multilineage hematopoiesis from immature human cells engrafted in SCID mice. Science. 1992;255:1137–41. 1372131 10.1126/science.1372131

[84] Shultz LD, et al. Multiple defects in innate and adaptive immunologic function in NOD/LtSz-scid mice. J Immunol. 1995;154:180–91. 7995938

[85] Takenaka K, et al. Polymorphism in Sirpa modulates engraftment of human hematopoietic stem cells. Nat Immunol. 2007;8:1313–23. 17982459 10.1038/ni1527

[86] Hesselton RM, et al. High levels of human peripheral blood mononuclear cell engraftment and enhanced susceptibility to human immunodeficiency virus type 1 infection in NOD/LtSz-scid/scid mice. J Infect Dis. 1995;172:974–82. 7561218 10.1093/infdis/172.4.974

[87] Ito M, et al. NOD/SCID/gamma(c)(null) mouse: an excellent recipient mouse model for engraftment of human cells. Blood. 2002;100:3175–82. 12384415 10.1182/blood-2001-12-0207

[88] Traggiai E, et al. Development of a human adaptive immune system in cord blood cell-transplanted mice. Science. 2004;304:104–7. 15064419 10.1126/science.1093933

[89] Shultz LD, et al. Human lymphoid and myeloid cell development in NOD/LtSz-scid IL2R gamma null mice engrafted with mobilized human hemopoietic stem cells. J Immunol. 2005;174:6477–89. 15879151 10.4049/jimmunol.174.10.6477

[90] Ishikawa F, et al. Development of functional human blood and immune systems in NOD/SCID/IL2 receptor {gamma} chain(null) mice. Blood. 2005;106:1565–73. 15920010 10.1182/blood-2005-02-0516 PMC1895228

[91] Brehm MA, et al. Parameters for establishing humanized mouse models to study human immunity: analysis of human hematopoietic stem cell engraftment in three immunodeficient strains of mice bearing the IL2rgamma(null) mutation. Clin Immunol. 2010;135:84–98. 20096637 10.1016/j.clim.2009.12.008 PMC2835837

[92] King MA, et al. Human peripheral blood leucocyte non-obese diabetic-severe combined immunodeficiency interleukin-2 receptor gamma chain gene mouse model of xenogeneic graft-versus-host-like disease and the role of host major histocompatibility complex. Clin Exp Immunol. 2009;157:104–18. 19659776 10.1111/j.1365-2249.2009.03933.x PMC2710598

[93] Nagatani M, et al. Comparison of biological features between severely immuno-deficient NOD/Shi-scid Il2rg(null) and NOD/LtSz-scid Il2rg(null) mice. Exp Anim. 2019;68:471–82. 31118345 10.1538/expanim.19-0024 PMC6842799

[94] Sondergaard H, Kvist PH, Haase C. Human T cells depend on functional calcineurin, tumour necrosis factor-alpha and CD80/CD86 for expansion and activation in mice. Clin Exp Immunol. 2013;172:300–10. 23574326 10.1111/cei.12051 PMC3628332

[95] Ehx G, et al. Xenogeneic graft-versus-host disease in humanized NSG and NSG-HLA-A2/HHD mice. Front Immunol. 2018;9:1943. 30214443 10.3389/fimmu.2018.01943 PMC6125392

[96] Covassin L, et al. Human peripheral blood CD4 T cell-engrafted non-obese diabetic-scid IL2rgamma(null) H2-Ab1 (tm1Gru) tg (human leucocyte antigen D-related 4) mice: a mouse model of human allogeneic graft-versus-host disease. Clin Exp Immunol. 2011;166:269–80. 21985373 10.1111/j.1365-2249.2011.04462.x PMC3219902

[97] Zeng Y, et al. Creation of an immunodeficient HLA-transgenic mouse (HUMAMICE) and functional validation of human immunity after transfer of HLA-matched human cells. PLoS ONE. 2017;12:e0173754. 28399128 10.1371/journal.pone.0173754 PMC5388326

[98] King M, et al. A new Hu-PBL model for the study of human islet alloreactivity based on NOD-scid mice bearing a targeted mutation in the IL-2 receptor gamma chain gene. Clin Immunol. 2008;126:303–14. 18096436 10.1016/j.clim.2007.11.001

[99] Chun S, et al. Double-filtered leukoreduction as a method for risk reduction of transfusion-associated graft-versus-host disease. PLoS ONE. 2020;15:e0229724. 32214402 10.1371/journal.pone.0229724 PMC7098637

[100] Bruck F, et al. Impact of bone marrow-derived mesenchymal stromal cells on experimental xenogeneic graft-versus-host disease. Cytotherapy. 2013;15:267–79. 23265769 10.1016/j.jcyt.2012.09.003

[101] Ito R, et al. Highly sensitive model for xenogenic GVHD using severe immunodeficient NOG mice. Transplantation. 2009;87:1654–8. 19502956 10.1097/TP.0b013e3181a5cb07

[102] Ali N, et al. Xenogeneic graft-versus-host-disease in NOD-scid IL-2Rgammanull mice display a T-effector memory phenotype. PLoS ONE. 2012;7:e44219. 22937164 10.1371/journal.pone.0044219 PMC3429415

[103] Ehx G, et al. Azacytidine prevents experimental xenogeneic graft-versus-host disease without abrogating graft-versus-leukemia effects. Oncoimmunology. 2017;6:e1314425. 28638744 10.1080/2162402X.2017.1314425 PMC5467988

[104] Kawasaki Y, et al. Comprehensive Analysis of the activation and Proliferation Kinetics and Effector functions of Human lymphocytes, and Antigen Presentation Capacity of Antigen-presenting cells in Xenogeneic graft-versus-host disease. Biol Blood Marrow Transpl. 2018;24:1563–74. 10.1016/j.bbmt.2018.04.016 29678638

[105] Geraghty NJ, et al. Increased splenic human CD4(+):CD8(+) T cell ratios, serum human interferon-γ and intestinal human interleukin-17 are associated with clinical graft-versus-host disease in humanized mice. Transpl Immunol. 2019;54:38–46. 30743002 10.1016/j.trim.2019.02.003

[106] Gao C, et al. Cytotoxic T lymphocyte antigen-4 regulates development of xenogenic graft versus host disease in mice via modulation of host immune responses induced by changes in human T cell engraftment and gene expression. Clin Exp Immunol. 2021;206:422–38. 34487545 10.1111/cei.13659 PMC8561689

[107] Ito R, et al. A Novel Xenogeneic graft-versus-host Disease Model for investigating the pathological role of human CD4(+) or CD8(+) T cells using immunodeficient NOG mice. Am J Transpl. 2017;17:1216–28. 10.1111/ajt.14116 27862942

[108] Cao X, et al. Defective lymphoid development in mice lacking expression of the common cytokine receptor gamma chain. Immunity. 1995;2:223–38. 7697543 10.1016/1074-7613(95)90047-0

[109] Chappaz S, Finke D. The IL-7 signaling pathway regulates lymph node development independent of peripheral lymphocytes. J Immunol. 2010;184:3562–9. 20207995 10.4049/jimmunol.0901647

[110] Takahashi T, et al. Enhanced antibody responses in a Novel NOG Transgenic mouse with restored Lymph Node Organogenesis. Front Immunol. 2017;8:2017. 29387068 10.3389/fimmu.2017.02017 PMC5776085

[111] DiSanto JP, Muller W, Guy-Grand D, Fischer A, Rajewsky K. Lymphoid development in mice with a targeted deletion of the interleukin 2 receptor gamma chain. Proc Natl Acad Sci U S A. 1995;92:377–81. 7831294 10.1073/pnas.92.2.377 PMC42743

[112] Miller PH, et al. Enhanced normal short-term human myelopoiesis in mice engineered to express human-specific myeloid growth factors. Blood. 2013;121:e1–4. 23233660 10.1182/blood-2012-09-456566

[113] Iwabuchi R, et al. Introduction of human Flt3-L and GM-CSF into Humanized mice enhances the reconstitution and maturation of myeloid dendritic cells and the development of Foxp3(+)CD4(+) T cells. Front Immunol. 2018;9:1042. 29892279 10.3389/fimmu.2018.01042 PMC5985304

[114] Ding Y, et al. FLT3-ligand treatment of humanized mice results in the generation of large numbers of CD141 + and CD1c + dendritic cells in vivo. J Immunol. 2014;192:1982–9. 24453245 10.4049/jimmunol.1302391

[115] Kennedy MK, et al. Reversible defects in natural killer and memory CD8 T cell lineages in interleukin 15-deficient mice. J Exp Med. 2000;191:771–80. 10704459 10.1084/jem.191.5.771 PMC2195858

[116] Huntington ND, et al. IL-15 trans-presentation promotes human NK cell development and differentiation in vivo. J Exp Med. 2009;206:25–34. 19103877 10.1084/jem.20082013 PMC2626663

[117] Fehniger TA, Caligiuri MA. Interleukin 15: biology and relevance to human disease. Blood. 2001;97:14–32. 11133738 10.1182/blood.v97.1.14

[118] Seay K, et al. In vivo activation of Human NK cells by treatment with an Interleukin-15 superagonist potently inhibits Acute in vivo HIV-1 infection in Humanized mice. J Virol. 2015;89:6264–74. 25833053 10.1128/JVI.00563-15 PMC4474292

[119] Koyama M, et al. Recipient nonhematopoietic antigen-presenting cells are sufficient to induce lethal acute graft-versus-host disease. Nat Med. 2011;18:135–42. 22127134 10.1038/nm.2597

[120] Koyama M, et al. MHC class II Antigen Presentation by the intestinal epithelium initiates graft-versus-host disease and is influenced by the Microbiota. Immunity. 2019;51:885–e898887. 31542340 10.1016/j.immuni.2019.08.011 PMC6959419

[121] Geraghty NJ, Watson D, Sluyter R. Pharmacological blockade of the CD39/CD73 pathway but not adenosine receptors augments disease in a humanized mouse model of graft-versus-host disease. Immunol Cell Biol. 2019;97:597–610. 30957314 10.1111/imcb.12251

[122] Cuthbertson P, et al. P2X7 receptor antagonism increases regulatory T cells and reduces clinical and histological graft-versus-host disease in a humanised mouse model. Clin Sci (Lond). 2021;135:495–513. 33463682 10.1042/CS20201352

[123] Geraghty NJ, et al. The P2X7 receptor antagonist Brilliant Blue G reduces serum human interferon-γ in a humanized mouse model of graft-versus-host disease. Clin Exp Immunol. 2017;190:79–95. 28665482 10.1111/cei.13005 PMC5588776

[124] Geraghty NJ, Watson D, Sluyter R. Long-term treatment with the P2X7 receptor antagonist Brilliant Blue G reduces liver inflammation in a humanized mouse model of graft-versus-host disease. Cell Immunol. 2019;336:12–9. 30545568 10.1016/j.cellimm.2018.12.001

[125] Beilhack A, et al. In vivo analyses of early events in acute graft-versus-host disease reveal sequential infiltration of T-cell subsets. Blood. 2005;106:1113–22. 15855275 10.1182/blood-2005-02-0509 PMC1895168

[126] Pektor S, et al. Using immuno-PET imaging to monitor kinetics of T cell-mediated inflammation and treatment efficiency in a humanized mouse model for GvHD. Eur J Nucl Med Mol Imaging. 2020;47:1314–25. 31471714 10.1007/s00259-019-04507-0

[127] Ehx G, et al. Comprehensive analysis of the immunomodulatory effects of rapamycin on human T cells in graft-versus-host disease prophylaxis. Am J Transpl. 2021;21:2662–74. 10.1111/ajt.16505 33512760

[128] Coman T, et al. Human CD4- invariant NKT lymphocytes regulate graft versus host disease. Oncoimmunology. 2018;7:e1470735. 30377560 10.1080/2162402X.2018.1470735 PMC6204997

[129] Silva IA, et al. Secondary lymphoid organs contribute to, but are not required for the induction of graft-versus-host responses following allogeneic bone marrow transplantation: a shifting paradigm for T cell allo-activation. Biol Blood Marrow Transpl. 2010;16:598–611. 10.1016/j.bbmt.2009.12.007 PMC3838892 20117226

[130] Anderson BE, et al. Effects of donor T-cell trafficking and priming site on graft-versus-host disease induction by naive and memory phenotype CD4 T cells. Blood. 2008;111:5242–51. 18285547 10.1182/blood-2007-09-107953 PMC2384145

[131] Feuerer M, et al. Bone marrow as a priming site for T-cell responses to blood-borne antigen. Nat Med. 2003;9:1151–7. 12910264 10.1038/nm914

[132] Wuensch SA, Pierce RH, Crispe IN. Local intrahepatic CD8 + T cell activation by a non-self-antigen results in full functional differentiation. J Immunol. 2006;177:1689–97. 16849478 10.4049/jimmunol.177.3.1689

[133] Moyron-Quiroz JE, et al. Role of inducible bronchus associated lymphoid tissue (iBALT) in respiratory immunity. Nat Med. 2004;10:927–34. 15311275 10.1038/nm1091

[134] Katz H, Victor L, Guinet E, Nouri-Shirazi M. Human T cells show plasticity for direct recognition of xenogeneic dendritic cells. Immunol Lett. 2022;248:90–5. 35753524 10.1016/j.imlet.2022.06.011

[135] Brehm MA, et al. Lack of acute xenogeneic graft- versus-host disease, but retention of T-cell function following engraftment of human peripheral blood mononuclear cells in NSG mice deficient in MHC class I and II expression. Faseb j. 2019;33:3137–51. 30383447 10.1096/fj.201800636R PMC6404556

[136] Collins FS, Mansoura MK. The Human Genome Project. Revealing the shared inheritance of all humankind. Cancer. 2001;91:221–5. 11148583 10.1002/1097-0142(20010101)91:1+<221::aid-cncr8>3.3.co;2-0

[137] Makałowski W, Zhang J, Boguski MS. Comparative analysis of 1196 orthologous mouse and human full-length mRNA and protein sequences. Genome Res. 1996;6:846–57. 8889551 10.1101/gr.6.9.846

[138] Meier JA, et al. T cell repertoire evolution after allogeneic bone marrow transplantation: an organizational perspective. Biol Blood Marrow Transpl. 2019;25:868–82. 10.1016/j.bbmt.2019.01.021 PMC6645918 30677510

[139] Abdul Razzaq B, et al. Dynamical System modeling to simulate donor T cell response to whole Exome sequencing-derived recipient peptides demonstrates different Alloreactivity potential in HLA-Matched and -mismatched donor-recipient pairs. Biol Blood Marrow Transpl. 2016;22:850–61. 10.1016/j.bbmt.2015.11.1103 PMC4955725 26688192

[140] Goel M, et al. Potential of TCR sequencing in graft-versus-host disease. Bone Marrow Transpl. 2023;58:239–46. 10.1038/s41409-022-01885-2 PMC10005964 36477111

[141] Kanakry CG et al. Origin and evolution of the T cell repertoire after posttransplantation cyclophosphamide. JCI Insight 1 (2016). 10.1172/jci.insight.86252 PMC4874509 27213183

[142] Trofimov A, et al. Two types of human TCR differentially regulate reactivity to self and non-self antigens. iScience. 2022;25:104968. 36111255 10.1016/j.isci.2022.104968 PMC9468382

[143] Rudd BD, Neonatal T, Cells. A reinterpretation. Annu Rev Immunol. 2020;38:229–47. 31928469 10.1146/annurev-immunol-091319-083608 PMC7369171

[144] Ritacco C, et al. Post-transplant cyclophosphamide prevents xenogeneic graft-versus-host disease while depleting proliferating regulatory T cells. iScience. 2023;26:106085. 36843851 10.1016/j.isci.2023.106085 PMC9947306

[145] DeZern AE, et al. Relationship of donor age and relationship to outcomes of haploidentical transplantation with posttransplant cyclophosphamide. Blood Adv. 2021;5:1360–8. 33661299 10.1182/bloodadvances.2020003922 PMC7948266

[146] Mehta RS et al. Impact of Donor Age in Haploidentical-Post-Transplantation Cyclophosphamide versus Matched Unrelated Donor Post-Transplantation Cyclophosphamide Hematopoietic Stem Cell Transplantation in Patients with Acute Myeloid Leukemia. *Transplant Cell Ther* 29, 377.e371-377.e377 (2023). 10.1016/j.jtct.2023.03.028 PMC10239355 36990221

[147] Martin PJ, et al. Genome-wide minor histocompatibility matching as related to the risk of graft-versus-host disease. Blood. 2017;129:791–8. 27872059 10.1182/blood-2016-09-737700 PMC5301826

[148] Fathman CG, Lineberry NB. Molecular mechanisms of CD4 + T-cell anergy. Nat Rev Immunol. 2007;7:599–609. 17612584 10.1038/nri2131

[149] Kumar S, Leigh ND, Cao X. The Role of Co-stimulatory/Co-inhibitory signals in Graft-vs.-Host disease. Front Immunol. 2018;9:3003. 30627129 10.3389/fimmu.2018.03003 PMC6309815

[150] Blazar BR, et al. Infusion of anti-B7.1 (CD80) and anti-B7.2 (CD86) monoclonal antibodies inhibits murine graft-versus-host disease lethality in part via direct effects on CD4 + and CD8 + T cells. J Immunol. 1996;157:3250–9. 8871619

[151] Yu XZ, Martin PJ, Anasetti C. Role of CD28 in acute graft-versus-host disease. Blood. 1998;92:2963–70. 9763584

[152] Koura DT, et al. In vivo T cell costimulation blockade with abatacept for acute graft-versus-host disease prevention: a first-in-disease trial. Biol Blood Marrow Transpl. 2013;19:1638–49. 10.1016/j.bbmt.2013.09.003 24047754

[153] Watkins B, et al. Phase II trial of Costimulation Blockade with Abatacept for Prevention of Acute GVHD. J Clin Oncol. 2021;39:1865–77. 33449816 10.1200/JCO.20.01086 PMC8260909

[154] Freeman GJ, et al. Murine B7-2, an alternative CTLA4 counter-receptor that costimulates T cell proliferation and interleukin 2 production. J Exp Med. 1993;178:2185–92. 7504059 10.1084/jem.178.6.2185 PMC2191273

[155] Burlion A, Brunel S, Petit NY, Olive D, Marodon G. Targeting the human T-Cell Inducible COStimulator Molecule with a monoclonal antibody prevents graft-vs-host disease and preserves graft vs leukemia in a xenograft murine model. Front Immunol. 2017;8:756. 28713380 10.3389/fimmu.2017.00756 PMC5491549

[156] Al-Khami AA, et al. Pharmacologic properties and preclinical activity of Sasanlimab, a high-affinity Engineered Anti-human PD-1 antibody. Mol Cancer Ther. 2020;19:2105–16. 32847983 10.1158/1535-7163.MCT-20-0093

[157] Hatano R, et al. Prevention of acute graft-versus-host disease by humanized anti-CD26 monoclonal antibody. Br J Haematol. 2013;162:263–77. 23692598 10.1111/bjh.12378

[158] Gao Y, et al. Daratumumab prevents experimental Xenogeneic graft-versus-host disease by skewing proportions of T cell functional subsets and inhibiting T cell activation and Migration. Front Immunol. 2021;12:785774. 34987512 10.3389/fimmu.2021.785774 PMC8720868

[159] Blazar BR, et al. Blockade of programmed death-1 engagement accelerates graft-versus-host disease lethality by an IFN-gamma-dependent mechanism. J Immunol. 2003;171:1272–7. 12874215 10.4049/jimmunol.171.3.1272

[160] Farag SS, et al. Dipeptidyl Peptidase 4 inhibition for Prophylaxis of Acute Graft-versus-host disease. N Engl J Med. 2021;384:11–9. 33406328 10.1056/NEJMoa2027372 PMC7845486

[161] Nikolaenko L, et al. Graft-versus-host disease in multiple myeloma patients treated with Daratumumab after allogeneic transplantation. Clin Lymphoma Myeloma Leuk. 2020;20:407–14. 32249196 10.1016/j.clml.2020.01.010 PMC9009296

[162] Vella AT, Dow S, Potter TA, Kappler J, Marrack P. Cytokine-induced survival of activated T cells in vitro and in vivo. Proc Natl Acad Sci U S A. 1998;95:3810–5. 9520449 10.1073/pnas.95.7.3810 PMC19919

[163] Moriggl R, et al. Stat5 is required for IL-2-induced cell cycle progression of peripheral T cells. Immunity. 1999;10:249–59. 10072077 10.1016/s1074-7613(00)80025-4

[164] Welte T, et al. STAT5 interaction with the T cell receptor complex and stimulation of T cell proliferation. Science. 1999;283:222–5. 9880255 10.1126/science.283.5399.222

[165] De Bock M, et al. Kinetics of IL-7 and IL-15 levels after allogeneic peripheral blood stem cell transplantation following nonmyeloablative conditioning. PLoS ONE. 2013;8:e55876. 23437070 10.1371/journal.pone.0055876 PMC3578874

[166] Tan JT, et al. IL-7 is critical for homeostatic proliferation and survival of naive T cells. Proc Natl Acad Sci U S A. 2001;98:8732–7. 11447288 10.1073/pnas.161126098 PMC37504

[167] Tan JT, et al. Interleukin (IL)-15 and IL-7 jointly regulate homeostatic proliferation of memory phenotype CD8 + cells but are not required for memory phenotype CD4 + cells. J Exp Med. 2002;195:1523–32. 12070280 10.1084/jem.20020066 PMC2193564

[168] Kieper WC, Jameson SC. Homeostatic expansion and phenotypic conversion of naïve T cells in response to self peptide/MHC ligands. Proc Natl Acad Sci U S A. 1999;96:13306–11. 10557316 10.1073/pnas.96.23.13306 PMC23943

[169] Kieper WC, et al. Recent immune status determines the source of antigens that drive homeostatic T cell expansion. J Immunol. 2005;174:3158–63. 15749843 10.4049/jimmunol.174.6.3158

[170] Murali-Krishna K, Ahmed R. Cutting edge: naive T cells masquerading as memory cells. J Immunol. 2000;165:1733–7. 10925249 10.4049/jimmunol.165.4.1733

[171] Goldrath AW, Bevan MJ. Low-affinity ligands for the TCR drive proliferation of mature CD8 + T cells in lymphopenic hosts. Immunity. 1999;11:183–90. 10485653 10.1016/s1074-7613(00)80093-x PMC2789737

[172] Barata JT, et al. Molecular and functional evidence for activity of murine IL-7 on human lymphocytes. Exp Hematol. 2006;34:1133–42. 16939806 10.1016/j.exphem.2006.05.001

[173] Coppin E, et al. Enhanced differentiation of functional human T cells in NSGW41 mice with tissue-specific expression of human interleukin-7. Leukemia. 2021;35:3561–7. 33976371 10.1038/s41375-021-01259-5 PMC8632686

[174] van Lent AU, et al. IL-7 enhances thymic human T cell development in human immune system Rag2-/-IL-2Rgammac-/- mice without affecting peripheral T cell homeostasis. J Immunol. 2009;183:7645–55. 19923447 10.4049/jimmunol.0902019

[175] Hara T, et al. Identification of IL-7-producing cells in primary and secondary lymphoid organs using IL-7-GFP knock-in mice. J Immunol. 2012;189:1577–84. 22786774 10.4049/jimmunol.1200586

[176] Onder L, et al. IL-7-producing stromal cells are critical for lymph node remodeling. Blood. 2012;120:4675–83. 22955921 10.1182/blood-2012-03-416859 PMC3952724

[177] Knop L, et al. IL-7 derived from lymph node fibroblastic reticular cells is dispensable for naive T cell homeostasis but crucial for central memory T cell survival. Eur J Immunol. 2020;50:846–57. 32043573 10.1002/eji.201948368

[178] Khosravi-Maharlooei M, et al. Rapid thymectomy of NSG mice to analyze the role of native and grafted thymi in humanized mice. Eur J Immunol. 2020;50:138–41. 31583677 10.1002/eji.201948205 PMC6940512

[179] van Ewijk W, Holländer G, Terhorst C, Wang B. Stepwise development of thymic microenvironments in vivo is regulated by thymocyte subsets. Development. 2000;127:1583–91. 10725235 10.1242/dev.127.8.1583

[180] Abraham S, et al. Long-term engraftment of human natural T regulatory cells in NOD/SCID IL2rgammac(null) mice by expression of human IL-2. PLoS ONE. 2012;7:e51832. 23272176 10.1371/journal.pone.0051832 PMC3525660

[181] Landwehr-Kenzel S, et al. Cyclosporine A but not corticosteroids Support efficacy of Ex vivo expanded, Adoptively Transferred Human tregs in GvHD. Front Immunol. 2021;12:716629. 34707604 10.3389/fimmu.2021.716629 PMC8543016

[182] Hippen KL, et al. Blocking IL-21 signaling ameliorates xenogeneic GVHD induced by human lymphocytes. Blood. 2012;119:619–28. 22077059 10.1182/blood-2011-07-368027 PMC3257019

[183] Zumwalde NA, Gumperz JE. Modeling human antitumor responses in vivo using umbilical cord blood-engrafted mice. Front Immunol. 2018;9:54. 29434589 10.3389/fimmu.2018.00054 PMC5790779

[184] Morillon YM 2nd, Sabzevari A, Schlom J, Greiner JW. The development of next-generation PBMC Humanized mice for Preclinical Investigation of Cancer Immunotherapeutic agents. Anticancer Res. 2020;40:5329–41. 10.21873/anticanres.14540 PMC8344070 32988851

[185] Hess NJ, Brown ME, Capitini CM. GVHD Pathogenesis, Prevention and Treatment: lessons from Humanized Mouse Transplant models. Front Immunol. 2021;12:723544. 34394131 10.3389/fimmu.2021.723544 PMC8358790

[186] Monti P, et al. Islet transplantation in patients with autoimmune diabetes induces homeostatic cytokines that expand autoreactive memory T cells. J Clin Invest. 2008;118:1806–14. 18431516 10.1172/JCI35197 PMC2323193

[187] Itamura H, et al. Pharmacological MEK inhibition promotes polyclonal T-cell reconstitution and suppresses xenogeneic GVHD. Cell Immunol. 2021;367:104410. 34274730 10.1016/j.cellimm.2021.104410

[188] Gregoire-Gauthier J, et al. Use of immunoglobulins in the prevention of GvHD in a xenogeneic NOD/SCID/γc- mouse model. Bone Marrow Transpl. 2012;47:439–50. 10.1038/bmt.2011.93 21572464

[189] Delens L, et al. In Vitro Th17-Polarized human CD4(+) T cells exacerbate Xenogeneic Graft-versus-host disease. Biol Blood Marrow Transpl. 2019;25:204–15. 10.1016/j.bbmt.2018.10.007 30326279

[190] Hannon M, et al. Infusion of clinical-grade enriched regulatory T cells delays experimental xenogeneic graft-versus-host disease. Transfusion. 2014;54:353–63. 23772685 10.1111/trf.12279

[191] Gattinoni L, Klebanoff CA, Restifo NP. Paths to stemness: building the ultimate antitumour T cell. Nat Rev Cancer. 2012;12:671–84. 22996603 10.1038/nrc3322 PMC6352980

[192] Koch S, et al. Multiparameter flow cytometric analysis of CD4 and CD8 T cell subsets in young and old people. Immun Ageing. 2008;5:6. 18657274 10.1186/1742-4933-5-6 PMC2515281

[193] Li M, et al. Age related human T cell subset evolution and senescence. Immun Ageing. 2019;16:24. 31528179 10.1186/s12979-019-0165-8 PMC6739976

[194] Kashiwagi H, et al. High-progesterone environment preserves T cell competency by evading glucocorticoid effects on immune regulation. Front Immunol. 2022;13:1000728. 36203559 10.3389/fimmu.2022.1000728 PMC9530059

[195] Hashimoto H, et al. Removal of CD276(+) cells from haploidentical memory T-cell grafts significantly lowers the risk of GVHD. Bone Marrow Transpl. 2021;56:2336–54. 10.1038/s41409-021-01307-9 PMC8486669 33976380

[196] Kueberuwa G, et al. CCR7(+) selected gene-modified T cells maintain a central memory phenotype and display enhanced persistence in peripheral blood in vivo. J Immunother Cancer. 2017;5:14. 28239467 10.1186/s40425-017-0216-7 PMC5319186

[197] Bleakley M, et al. Naive T-Cell depletion to prevent chronic graft-versus-host disease. J Clin Oncol. 2022;40:1174–85. 35007144 10.1200/JCO.21.01755 PMC8987226

[198] Anderson BE, et al. Memory CD4 + T cells do not induce graft-versus-host disease. J Clin Invest. 2003;112:101–8. 12840064 10.1172/JCI17601 PMC162285

[199] Ito R, et al. Exacerbation of pathogenic Th17-cell-mediated cutaneous graft-versus-host-disease in human IL-1β and IL-23 transgenic humanized mice. Biochem Biophys Res Commun. 2019;516:480–5. 31230747 10.1016/j.bbrc.2019.06.094

[200] Asakura S, et al. Alloantigen expression on non-hematopoietic cells reduces graft-versus-leukemia effects in mice. J Clin Invest. 2010;120:2370–8. 20530875 10.1172/JCI39165 PMC2898583

[201] Simonetta F, et al. Dynamics of expression of programmed cell death Protein-1 (PD-1) on T cells after allogeneic hematopoietic stem cell transplantation. Front Immunol. 2019;10:1034. 31156625 10.3389/fimmu.2019.01034 PMC6531929

[202] Bengsch B, et al. Bioenergetic insufficiencies due to metabolic alterations regulated by the inhibitory receptor PD-1 are an early driver of CD8(+) T cell exhaustion. Immunity. 2016;45:358–73. 27496729 10.1016/j.immuni.2016.07.008 PMC4988919

[203] Miller BC, et al. Subsets of exhausted CD8(+) T cells differentially mediate tumor control and respond to checkpoint blockade. Nat Immunol. 2019;20:326–36. 30778252 10.1038/s41590-019-0312-6 PMC6673650

[204] Saeidi A, et al. T-Cell exhaustion in chronic infections: reversing the state of exhaustion and reinvigorating Optimal Protective Immune responses. Front Immunol. 2018;9:2569. 30473697 10.3389/fimmu.2018.02569 PMC6237934

[205] Alhaj Hussen K, et al. CD4(+)CD8(+) T-Lymphocytes in Xenogeneic and Human Graft-versus-host disease. Front Immunol. 2020;11:579776. 33329550 10.3389/fimmu.2020.579776 PMC7732609

[206] Pyo KH, et al. Promising preclinical platform for evaluation of immuno-oncology drugs using Hu-PBL-NSG lung cancer models. Lung Cancer. 2019;127:112–21. 30642538 10.1016/j.lungcan.2018.11.035

[207] Szklarczyk D, et al. The STRING database in 2023: protein-protein association networks and functional enrichment analyses for any sequenced genome of interest. Nucleic Acids Res. 2023;51:D638–46. 36370105 10.1093/nar/gkac1000 PMC9825434

[208] Hempel L, et al. High interleukin-10 serum levels are associated with fatal outcome in patients after bone marrow transplantation. Bone Marrow Transpl. 1997;20:365–8. 10.1038/sj.bmt.1700902 9339750

[209] Miura Y, et al. Cytokine and chemokine profiles in autologous graft-versus-host disease (GVHD): interleukin 10 and interferon gamma may be critical mediators for the development of autologous GVHD. Blood. 2002;100:2650–8. 12239181 10.1182/blood-2002-01-0176

[210] Blazar BR, et al. Interleukin-10 dose-dependent regulation of CD4 + and CD8 + T cell-mediated graft-versus-host disease. Transplantation. 1998;66:1220–9. 9825821 10.1097/00007890-199811150-00018

[211] Abraham S, Choi JG, Ye C, Manjunath N, Shankar P. IL-10 exacerbates xenogeneic GVHD by inducing massive human T cell expansion. Clin Immunol. 2015;156:58–64. 25463432 10.1016/j.clim.2014.11.004 PMC4310723

[212] Hess NJ, et al. Inflammatory CD4/CD8 double-positive human T cells arise from reactive CD8 T cells and are sufficient to mediate GVHD pathology. Sci Adv. 2023;9:eadf0567. 36961891 10.1126/sciadv.adf0567 PMC10038349

[213] Betts BC et al. Targeting Aurora kinase A and JAK2 prevents GVHD while maintaining Treg and antitumor CTL function. Sci Transl Med 9 (2017). 10.1126/scitranslmed.aai8269 PMC6368389 28077684

[214] Boucault L, et al. Transient antibody targeting of CD45RC inhibits the development of graft-versus-host disease. Blood Adv. 2020;4:2501–15. 32511714 10.1182/bloodadvances.2020001688 PMC7284095

[215] Cuthbertson P et al. Post-transplant Cyclophosphamide Combined with Brilliant Blue G reduces graft-versus-host disease without compromising graft-versus-leukaemia immunity in Humanised mice. Int J Mol Sci 25 (2024). 10.3390/ijms25031775 PMC10855770 38339054

[216] Neidemire-Colley L, et al. CRISPR/Cas9 deletion of MIR155HG in human T cells reduces incidence and severity of acute GVHD in a xenogeneic model. Blood Adv. 2024;8:947–58. 38181781 10.1182/bloodadvances.2023010570 PMC10877121

[217] Sligar C, et al. Tocilizumab increases regulatory T cells, reduces natural killer cells and delays graft-versus-host disease development in humanized mice treated with post-transplant cyclophosphamide. Immunol Cell Biol. 2023;101:639–56. 37191045 10.1111/imcb.12652

[218] Adhikary SR, et al. Post-transplant cyclophosphamide limits reactive donor T cells and delays the development of graft-versus-host disease in a humanized mouse model. Immunology. 2021;164:332–47. 34021907 10.1111/imm.13374 PMC8442241

[219] Mashima K, et al. Comparison of alemtuzumab, anti-thymocyte globulin, and post-transplant cyclophosphamide for graft-versus-host disease and graft-versus-leukemia in murine models. PLoS ONE. 2021;16:e0245232. 33428661 10.1371/journal.pone.0245232 PMC7799789

[220] Kanakry CG, et al. Aldehyde dehydrogenase expression drives human regulatory T cell resistance to posttransplantation cyclophosphamide. Sci Transl Med. 2013;5:211ra157. 24225944 10.1126/scitranslmed.3006960 PMC4155575

[221] Norona J, et al. Glucagon-like peptide 2 for intestinal stem cell and Paneth cell repair during graft-versus-host disease in mice and humans. Blood. 2020;136:1442–55. 32542357 10.1182/blood.2020005957 PMC7498363

[222] Huang H, Zheng W, Lin M, Fu J, Zhang R. [Prophylaxis of acute graft-versus-host disease after allogeneic bone marrow transplantation by mycophenolate mofetil in a murine model]. Zhonghua Xue Ye Xue Za Zhi. 2002;23:191–3. 12133455

[223] Li X, et al. Cyclosporine A regulates PMN-MDSCs viability and function through MPTP in acute GVHD: old medication, new target. Transpl Cell Ther. 2022;28:e411411–9. 10.1016/j.jtct.2022.04.010 35430420

[224] Al-Homsi AS, et al. Post-transplantation Cyclophosphamide and Ixazomib Combination rescues mice subjected to experimental graft-versus-host disease and is Superior to either Agent alone. Biol Blood Marrow Transpl. 2017;23:255–61. 10.1016/j.bbmt.2016.11.015 27888016

[225] Baron F, et al. Thinking out of the box–new approaches to controlling GVHD. Curr Hematol Malig Rep. 2014;9:73–84. 24390548 10.1007/s11899-013-0187-9

[226] Schmidt A, Oberle N, Krammer PH. Molecular mechanisms of treg-mediated T cell suppression. Front Immunol. 2012;3:51. 22566933 10.3389/fimmu.2012.00051 PMC3341960

[227] Burchill MA, Yang J, Vogtenhuber C, Blazar BR, Farrar MA. IL-2 receptor beta-dependent STAT5 activation is required for the development of Foxp3 + regulatory T cells. J Immunol. 2007;178:280–90. 17182565 10.4049/jimmunol.178.1.280

[228] Rodriguez-Perea AL, Arcia ED, Rueda CM, Velilla PA. Phenotypical characterization of regulatory T cells in humans and rodents. Clin Exp Immunol. 2016;185:281–91. 27124481 10.1111/cei.12804 PMC4991523

[229] Ehx G, Hannon M, Beguin Y, Humblet-Baron S, Baron F. Validation of a multicolor staining to monitor phosphoSTAT5 levels in regulatory T-cell subsets. Oncotarget. 2015;6:43255–66. 26657728 10.18632/oncotarget.6486 PMC4791230

[230] Ritacco C, et al. High proportion of terminally differentiated regulatory T cells after allogeneic hematopoietic stem cell transplantation. Bone Marrow Transpl. 2021;56:1828–41. 10.1038/s41409-021-01221-0 33664462

[231] Hori S, Nomura T, Sakaguchi S. Control of regulatory T cell development by the transcription factor Foxp3. Science. 2003;299:1057–61. 12522256 10.1126/science.1079490

[232] Hoffmann P, Ermann J, Edinger M, Fathman CG, Strober S. Donor-type CD4(+)CD25(+) regulatory T cells suppress lethal acute graft-versus-host disease after allogeneic bone marrow transplantation. J Exp Med. 2002;196:389–99. 12163567 10.1084/jem.20020399 PMC2193938

[233] Taylor PA, Lees CJ, Blazar BR. The infusion of ex vivo activated and expanded CD4(+)CD25(+) immune regulatory cells inhibits graft-versus-host disease lethality. Blood. 2002;99:3493–9. 11986199 10.1182/blood.v99.10.3493

[234] Trenado A, et al. Recipient-type specific CD4 + CD25 + regulatory T cells favor immune reconstitution and control graft-versus-host disease while maintaining graft-versus-leukemia. J Clin Invest. 2003;112:1688–96. 14660744 10.1172/JCI17702 PMC281639

[235] Koreth J, et al. Interleukin-2 and regulatory T cells in graft-versus-host disease. N Engl J Med. 2011;365:2055–66. 22129252 10.1056/NEJMoa1108188 PMC3727432

[236] Koreth J, et al. Efficacy, durability, and response predictors of low-dose interleukin-2 therapy for chronic graft-versus-host disease. Blood. 2016;128:130–7. 27073224 10.1182/blood-2016-02-702852 PMC4937358

[237] Kennedy-Nasser AA, et al. Ultra low-dose IL-2 for GVHD prophylaxis after allogeneic hematopoietic stem cell transplantation mediates expansion of regulatory T cells without diminishing antiviral and antileukemic activity. Clin Cancer Res. 2014;20:2215–25. 24573552 10.1158/1078-0432.CCR-13-3205 PMC3989436

[238] Parmar S, et al. Third-party umbilical cord blood-derived regulatory T cells prevent xenogenic graft-versus-host disease. Cytotherapy. 2014;16:90–100. 24480547 10.1016/j.jcyt.2013.07.009 PMC4124936

[239] Barreras H et al. Regulatory T Cell Amelioration of Graft-versus-Host Disease following Allogeneic/Xenogeneic Hematopoietic Stem Cell Transplantation Using Mobilized Mouse and Human Peripheral Blood Donors. *Transplant Cell Ther* 29, 341.e341-341.e349 (2023). 10.1016/j.jtct.2023.02.015 PMC10149591 36804930

[240] Cuende J, et al. Monoclonal antibodies against GARP/TGF-β1 complexes inhibit the immunosuppressive activity of human regulatory T cells in vivo. Sci Transl Med. 2015;7:284ra256. 10.1126/scitranslmed.aaa1983 25904740

[241] Courtois J, et al. Itacitinib prevents xenogeneic GVHD in humanized mice. Bone Marrow Transpl. 2021;56:2672–81. 10.1038/s41409-021-01363-1 PMC8563409 34172892

[242] Hu M, et al. Low-dose Interleukin-2 combined with Rapamycin Led to an expansion of CD4(+)CD25(+)FOXP3(+) Regulatory T cells and prolonged human islet allograft survival in Humanized mice. Diabetes. 2020;69:1735–48. 32381646 10.2337/db19-0525

[243] Trotta E, et al. A human anti-IL-2 antibody that potentiates regulatory T cells by a structure-based mechanism. Nat Med. 2018;24:1005–14. 29942088 10.1038/s41591-018-0070-2 PMC6398608

[244] Ferreira LMR, Muller YD, Bluestone JA, Tang Q. Next-generation regulatory T cell therapy. Nat Rev Drug Discov. 2019;18:749–69. 31541224 10.1038/s41573-019-0041-4 PMC7773144

[245] MacDonald KG, et al. Alloantigen-specific regulatory T cells generated with a chimeric antigen receptor. J Clin Invest. 2016;126:1413–24. 26999600 10.1172/JCI82771 PMC4811124

[246] Rui X, et al. Human OX40L-CAR-T(regs) target activated antigen-presenting cells and control T cell alloreactivity. Sci Transl Med. 2024;16:eadj9331. 39413160 10.1126/scitranslmed.adj9331 PMC11789419

[247] Yano H, et al. Human iPSC-derived CD4(+) Treg-like cells engineered with chimeric antigen receptors control GvHD in a xenograft model. Cell Stem Cell. 2024;31:795–e802796. 38848686 10.1016/j.stem.2024.05.004

[248] Proics E, et al. Preclinical assessment of antigen-specific chimeric antigen receptor regulatory T cells for use in solid organ transplantation. Gene Ther. 2023;30:309–22. 35931871 10.1038/s41434-022-00358-x PMC10113151

[249] Dominici M, et al. Minimal criteria for defining multipotent mesenchymal stromal cells. The International Society for Cellular Therapy position statement. Cytotherapy. 2006;8:315–7. 16923606 10.1080/14653240600855905

[250] Mattar P, Bieback K. Comparing the Immunomodulatory properties of Bone Marrow, adipose tissue, and Birth-Associated tissue mesenchymal stromal cells. Front Immunol. 2015;6:560. 26579133 10.3389/fimmu.2015.00560 PMC4630659

[251] Waldner M, et al. Characteristics and immunomodulating functions of adipose-derived and bone marrow-derived mesenchymal stem cells across defined human leukocyte Antigen barriers. Front Immunol. 2018;9:1642. 30087676 10.3389/fimmu.2018.01642 PMC6066508

[252] Le Blanc K, Mougiakakos D. Multipotent mesenchymal stromal cells and the innate immune system. Nat Rev Immunol. 2012;12:383–96. 22531326 10.1038/nri3209

[253] Ball LM, et al. Cotransplantation of ex vivo expanded mesenchymal stem cells accelerates lymphocyte recovery and may reduce the risk of graft failure in haploidentical hematopoietic stem-cell transplantation. Blood. 2007;110:2764–7. 17638847 10.1182/blood-2007-04-087056

[254] Ning H, et al. The correlation between cotransplantation of mesenchymal stem cells and higher recurrence rate in hematologic malignancy patients: outcome of a pilot clinical study. Leukemia. 2008;22:593–9. 18185520 10.1038/sj.leu.2405090

[255] Baron F, et al. Cotransplantation of mesenchymal stem cells might prevent death from graft-versus-host disease (GVHD) without abrogating graft-versus-tumor effects after HLA-mismatched allogeneic transplantation following nonmyeloablative conditioning. Biol Blood Marrow Transpl. 2010;16:838–47. 10.1016/j.bbmt.2010.01.011 20109568

[256] Kallekleiv M, Larun L, Bruserud Ø, Hatfield KJ. Co-transplantation of multipotent mesenchymal stromal cells in allogeneic hematopoietic stem cell transplantation: a systematic review and meta-analysis. Cytotherapy. 2016;18:172–85. 26794711 10.1016/j.jcyt.2015.11.010

[257] Girdlestone J, et al. Enhancement of the immunoregulatory potency of mesenchymal stromal cells by treatment with immunosuppressive drugs. Cytotherapy. 2015;17:1188–99. 26276002 10.1016/j.jcyt.2015.05.009

[258] Tobin LM, Healy ME, English K, Mahon BP. Human mesenchymal stem cells suppress donor CD4(+) T cell proliferation and reduce pathology in a humanized mouse model of acute graft-versus-host disease. Clin Exp Immunol. 2013;172:333–48. 23574329 10.1111/cei.12056 PMC3628335

[259] Tisato V, Naresh K, Girdlestone J, Navarrete C, Dazzi F. Mesenchymal stem cells of cord blood origin are effective at preventing but not treating graft-versus-host disease. Leukemia. 2007;21:1992–9. 17625609 10.1038/sj.leu.2404847

[260] Gregoire-Gauthier J, et al. Therapeutic efficacy of cord blood-derived mesenchymal stromal cells for the prevention of acute graft-versus-host disease in a xenogenic mouse model. Stem Cells Dev. 2012;21:1616–26. 21910645 10.1089/scd.2011.0413

[261] Jang YK, et al. Optimization of the therapeutic efficacy of human umbilical cord blood-mesenchymal stromal cells in an NSG mouse xenograft model of graft-versus-host disease. Cytotherapy. 2014;16:298–308. 24418403 10.1016/j.jcyt.2013.10.012

[262] Kim DS, et al. Application of human mesenchymal stem cells cultured in different oxygen concentrations for treatment of graft-versus-host disease in mice. Biomed Res. 2016;37:311–7. 27784875 10.2220/biomedres.37.311

[263] Amarnath S, et al. Bone marrow-derived mesenchymal stromal cells harness purinergenic signaling to tolerize human Th1 cells in vivo. Stem Cells. 2015;33:1200–12. 25532725 10.1002/stem.1934 PMC4376558

[264] Ma Y, et al. Human placenta-derived mesenchymal stem cells ameliorate GVHD by modulating Th17/Tr1 balance via expression of PD-L2. Life Sci. 2018;214:98–105. 30393022 10.1016/j.lfs.2018.10.061

[265] Grégoire C, et al. Comparison of mesenchymal stromal cells from different origins for the treatment of Graft-vs.-Host-disease in a Humanized Mouse Model. Front Immunol. 2019;10:619. 31001253 10.3389/fimmu.2019.00619 PMC6454068

[266] Yañez R, et al. Adipose tissue-derived mesenchymal stem cells have in vivo immunosuppressive properties applicable for the control of the graft-versus-host disease. Stem Cells. 2006;24:2582–91. 16873762 10.1634/stemcells.2006-0228

[267] Sudres M, et al. Bone marrow mesenchymal stem cells suppress lymphocyte proliferation in vitro but fail to prevent graft-versus-host disease in mice. J Immunol. 2006;176:7761–7. 16751424 10.4049/jimmunol.176.12.7761

[268] Demosthenous C et al. The role of myeloid-derived suppressor cells (MDSCs) in graft-versus-host disease (GVHD). J Clin Med 10 (2021). 10.3390/jcm10102050 PMC8150814 34064671

[269] Park MY, et al. GM-CSF promotes the expansion and differentiation of cord blood myeloid-derived suppressor cells, which attenuate Xenogeneic Graft-vs.-Host disease. Front Immunol. 2019;10:183. 30863394 10.3389/fimmu.2019.00183 PMC6399310

[270] Janikashvili N, et al. Efficiency of human monocyte-derived suppressor cell-based treatment in graft-versus-host disease prevention while preserving graft-versus-leukemia effect. Oncoimmunology. 2021;10:1880046. 33659098 10.1080/2162402X.2021.1880046 PMC7899641

[271] Gérard C, et al. Human monocyte-derived suppressor cell supernatant induces Immunoregulatory effects and mitigates xenoGvHD. Front Immunol. 2022;13:827712. 35345675 10.3389/fimmu.2022.827712 PMC8957111

[272] Matsuda M et al. Human NK cell development in hIL-7 and hIL-15 knockin NOD/SCID/IL2rgKO mice. Life Sci Alliance 2 (2019). 10.26508/lsa.201800195 PMC6445396 30936185

[273] Wunderlich M, et al. AML xenograft efficiency is significantly improved in NOD/SCID-IL2RG mice constitutively expressing human SCF, GM-CSF and IL-3. Leukemia. 2010;24:1785–8. 20686503 10.1038/leu.2010.158 PMC5439963

[274] Cimbro R, et al. IL-7 induces expression and activation of integrin α4β7 promoting naive T-cell homing to the intestinal mucosa. Blood. 2012;120:2610–9. 22896005 10.1182/blood-2012-06-434779 PMC3460683

[275] Noronha N, et al. Major multilevel molecular divergence between THP-1 cells from different biorepositories. Int J Cancer. 2020;147:2000–6. 32163592 10.1002/ijc.32967

